# Density Functional Theory Study of Iron–Oxygen Divacancies in Magnetite (Fe_3_O_4_) and Hematite (Fe_2_O_3_)

**DOI:** 10.1021/acs.jpcc.5c02852

**Published:** 2025-08-19

**Authors:** Shivani Srivastava, Blas Pedro Uberuaga, Mark Asta

**Affiliations:** † Department of Materials Science and Engineering, University of California, Berkeley, California 94720, United States; ‡ Materials Science and Technology Division, 5112 Los Alamos National Laboratory, Los Alamos, New Mexico 87544, United States; § Materials Sciences Division, Lawrence Berkeley National Laboratory, Berkeley, California 94720, United States

## Abstract

Density functional theory (DFT) calculations are employed to investigate the formation energies, charge redistribution, and binding energies of iron–oxygen divacancies in magnetite (Fe_3_O_4_) and hematite (Fe_2_O_3_). For magnetite, we focus on the low-temperature phase to explore variations with local environments. Building on previous DFT calculations of the variations in formation energies for oxygen vacancies with local charge and spin order in magnetite, we extend this analysis to include octahedral iron vacancies before analyzing the iron–oxygen divacancies. We also assessed the relative stability of iron–oxygen divacancies by comparing their formation energies with those of individual vacancies. Our findings reveal a significant energetic driving force for the formation of divacancy clusters, particularly in magnetite, where divacancies in the +1 charge state exhibit formation energies comparable to those of neutral iron vacancies under oxidizing conditions. In hematite, the results indicate a strong tendency for oxygen vacancies to bind to iron vacancies. These results highlight the significance of iron–oxygen vacancy complexes in the transport properties of iron oxides, with particular relevance to diffusion mechanisms under irradiation conditions.

## Graphical abstract

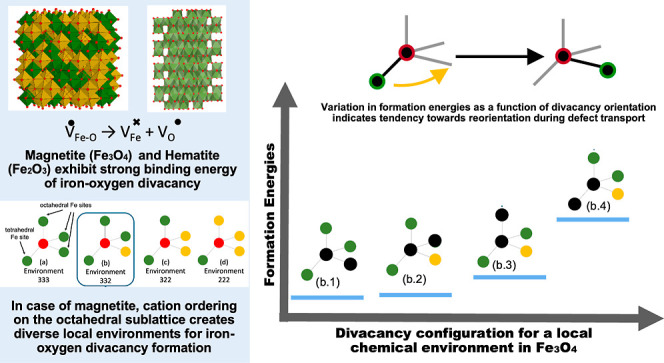

## Introduction

Understanding the thermodynamic properties of point defects in iron oxides is technologically relevant due to their presence in various applications, such as mixed oxide layers formed during iron corrosion in air or aqueous environments;^1–3^ catalysis for water–gas shift reactions, industrial wastewater decontamination, and surface-mediated reduction of uranyl;^4–7^ spintronic devices;^8,9^ etc. Due to their essential role in defining material properties for the aforementioned applications, point defects in iron oxides such as magnetite (Fe_3_O_4_) and hematite (Fe_2_O_3_) have been extensively investigated both experimentally^10–13^ and computationally.^14–23^ However, defect complexes such as cation–anion divacancies are comparatively less studied. These defect complexes could have a significant^24,25^ role in interpreting defect transport under irradiation conditions. In oxides exposed to such conditions, irradiation can initially knock cations and anions from their lattice sites, creating vacancies and interstitials, which can then relax back to an equilibrium structure. Previous work^24,25^ has demonstrated that these defects can sometimes form metastable clusters that have reduced mobility and therefore can take a long time to anneal or diffuse toward sinks such as grain boundaries and surfaces. A previous study on MgAl_2_O_4_, a spinel, using the speculatively parallel temperature accelerated dynamics (SpecTAD)^26^ simulations, provides some key insights in the form of the relative stability of two types of cation–anion vacancies in MgAl_2_O_4_: V_Mg_–V_O_ and V_Al_–V_O_ and the difference in their diffusion mechanism. For both of these divacancies, clustering of cation and anion vacancies reduces the mobility of point defects. Bound cation–anion divacancies are also found to be stable in other oxide systems, such as lead titanate (PbTiO_3_)^27^ and magnesium oxide.^28^

Due to the requirement of large supercell sizes to incorporate the resulting lattice distortions and changes in the local electronic structure, previous computational studies of divacancy defects in ionic materials usually employ classical potentials,^29^ which do not allow the ionic charge to change. As presented in later sections, in the case of magnetite, the resulting electron redistribution around even a simple complex such as an iron–oxygen divacancy can occur within a radius of 6–8 Å around the defect site and is accompanied by a change in the oxidation states of Fe^3+^ and Fe^2+^ ions. Specifically for the iron oxides studied in this paper, magnetite (Fe_3_O_4_) and hematite (Fe_2_O_3_), experimental studies have speculated about the existence of Fe–O divacancies to explain some of the observations. In tracer diffusion studies^30^ in magnetite, under oxygen-rich conditions, the minority defects responsible for oxygen transport are hypothesized to be Fe–O divacancies. In the case of hematite (Fe_2_O_3_), a recent study^31^ performed on irradiated hematite films points toward the possibility of strong interactions between defects in the cation and anion sublattices, which could lead to clustering and correlate the self-diffusion behavior of cation and anion defects.

We extend previous computational studies by employing density functional theory (DFT) to study Fe–O divacancy properties in low-temperature magnetite, where charge and spin ordering create diverse local atomic environments, and then to hematite, which exhibits no such charge ordering. In our previous work,^32^ we focused on oxygen vacancies and explored local-environment dependencies in the low-temperature monoclinic phase, where Fe^2+^ and Fe^3+^ ions exhibit long-range ordering over the octahedral sites of the inverse-spinel structure. The results and discussion presented are structured as follows. We begin with results on iron vacancies in monoclinic magnetite to explore the effects of local environments on charge redistribution and formation energies. The rationale for using this phase is detailed in our previous work.^32^ As in previous studies,^19,22^ neutral iron vacancies in the tetrahedral sublattice have higher formation energies than those in the octahedral sublattice by 1.13–2.2 eV, depending on the local environment. Hence, we focus on iron vacancies in octahedral sites for a detailed analysis. We then present formation energy, binding energy, and charge redistribution results for Fe–O divacancies in magnetite in various local environments, classified on the basis of the oxygen and iron vacancy sites involved. We end our analysis by providing results and a discussion on Fe–O divacancies in hematite (Fe_2_O_3_), another oxide found as a component of multioxide scales that can form upon oxidation of iron.^30^ The present work is focused on the energetics of divacancies and sets the stage for future work to compute migration energies for models of ionic transport.

## Computational Methods

DFT calculations for magnetite and hematite supercells were performed using the projector augmented wave (PAW) method^33,34^ within the VASP package.^35^ The Perdew–Burke–Ernzerhof (PBE) generalized gradient approximation (GGA)^36^ was employed, along with a Hubbard *U* correction for Fe 3d electrons, following Dudarev et al.’s approach.^37^ PBE-GGA pseudopotentials “Fe_pv” and “O” were used, including appropriate valence states for Fe and O. For capturing an appropriate localization of electrons, as described in our previous work,^32^ a Hubbard *U* correction of *U*_eff_ = 4.1 eV, using a rotationally invariant approach,^37^ was applied on the Fe d orbitals. While hybrid functionals such as HSE06 improve band gap predictions, their reliability in capturing the relative energetics of different iron oxides remains uncertain.^38^ Due to the high computational cost of hybrid functionals and the need to sample multiple defect configurations, we employed the GGA + *U* method in this work, balancing accuracy with feasibility. Hybrid functionals impact results primarily by changing electron and hole localization^39^ and by altering calculated chemical potentials for defect formation energy calculations. To address the latter, postprocessing correction schemes have been proposed to allow meaningful comparisons with experiments.^40^

For magnetite, a 600 eV plane-wave cutoff and gamma-point *k*-point sampling were used for bulk structure optimizations of the 224-atom monoclinic and 448-atom supercells.^32^ A reduced 450 eV cutoff and gamma-only sampling were used for relaxing monoclinic cells with iron vacancies. For divacancy defect calculations, a 448-atom supercell with long-range ordering similar to that of the 224-atom supercell was employed. Final energies were obtained using static calculations on relaxed structures with a 3 × 3 × 2 *k*-point mesh for the 224-atom cell and a 2 × 2 × 2 mesh for the 448-atom supercell, employing tetrahedron integration with Blöchl corrections.^41^ All relaxations were spin-polarized, with energy convergence set to 10^–4^ eV and force convergence set to 0.01 eV/Å.

The computational methods employed in hematite DFT simulations are the same as the published work on thermokinetics,^42^ on which this work is based. The choice of the same convergence parameters, pseudopotentials, and *U* values was made to compare the thermodynamics of the Fe–O vacancy cluster (referred to as divacancy) and the electron polaron to the published data on iron and oxygen vacancies. The chemical potential values and dielectric constants for the defect formation energy calculations are also consistent with this previous study.^42^ Formation energies of different charge states of the iron and oxygen vacancy values are taken from this previous work,^42^ and we have only performed the additional divacancy DFT calculations for this work.

For the *U* values mentioned above, the calculated band gap is approximately 1.1 eV for the low-temperature phase of magnetite and 2.24 eV for hematite, as reported in the referenced work. These values are used to define the range of Fermi energies in the formation energy plots, spanning from the valence band maximum (VBM) to the conduction band minimum (CBM), with the Fermi level referenced relative to the VBM.

## Defect Formation Energies

The formation energies were calculated as a function of charge state employing the traditional dilute-density, grand-canonical formalism and appropriate corrections for artifacts arising from electrostatic interaction of image charges due to finite supercell sizes.^43–47^ Specifically, the formation energy (*E*_form_) of a defect in charge state *q* is given as

1 $$E_{form}=E_{\mathrm{def}}-E_{bulk}-\sum_{n=1}\mu_{i}\delta n_{i}+\left(E_{f}+E_{VBM}\right)q+\delta E_{corrections}$$

where *E*_def_ is the total energy of the defect supercell, *E*_bulk_ is the reference total energy of the bulk supercell, μ is the chemical potential of atoms being added (δ*n* > 0) or removed (δ*n* < 0), *E*_f_ is the Fermi level referenced to valence band maxima (*E*_VBM_), and δ*E*_corrections_ are the finite supercell size corrections.^46^

The values of the supercell corrections (δ*E*_corrections_) in eq 1 were derived using the formalism proposed by Kumagai and Oba,^46^ which requires knowledge of the dielectric tensor. Similar to our previous work,^32^ the dielectric tensor was calculated using density functional perturbation theory (DFPT) as implemented in VASP^40,48^ using a smaller 14-atom cell model for magnetite,^49^ due to the high computational costs. The values in the anisotropic dielectric tensor vary between 13.19 and 19.76 for different crystallographic axes.

For the defect formation energy calculations of iron vacancies and Fe–O divacancies, we have used chemical potentials corresponding to the oxidizing limit given by the magnetite in equilibrium with hematite (Fe_2_O_3_). This value is obtained from the energies per formula unit for Fe_2_O_3_ and Fe_3_O_4_ as $\mu_{O}=3\times E_{Fe_{2}O_{3}}-2\times E_{Fe_{3}O_{4}}$ , where $E_{Fe_{3}O_{4}}$ was computed assuming the low-temperature monoclinic structure and $E_{Fe_{2}O_{3}}$ corresponds to Fe_2_O_3_ in a structure with *R*3̅*c* symmetry and antiferromagnetic magnetic ordering. Given the sensitivity of chemical potentials to the exchange–correlation functional, a posteriori corrections^50,51^ can be applied to defect formation energies. However, no such corrections are applied here, as the primary focus of this work is on studying relative formation energies and divacancy binding energies that are not affected by choice of chemical potentials.

Magnetite (Fe_3_O_4_) is known to exhibit orbital ordering,^52^ resulting in multiple local minima for each defect configuration due to strong coupling between orbital occupancy and structural distortions. Although multiple local orbital occupations were not systematically explored here, tests on selected defect configurations in our previous work^32^ involving constrained total magnetization and atomic position perturbations around the defects consistently converged to the same low-energy states. Strong antiferromagnetic coupling between octahedral and tetrahedral sublattices leads to a preferred spin state within the collinear DFT approximation, and self-consistent calculations reliably find stable minima. Due to computational cost, extensive sampling for all possible orbital configurations for each defect configuration was not feasible.

## Results

We present calculated results demonstrating that under oxidizing conditions in magnetite (Fe_3_O_4_), Fe–O divacancies exhibit formation energies comparable to those of iron vacancies and form strongly bound complexes. This study builds upon our previous work on oxygen vacancies in the low-temperature phase of magnetite.^32^ Additionally, we analyze the clustering behavior of iron and oxygen vacancies in hematite (Fe_2_O_3_) and find a significant energetic driving force for oxygen vacancies to bind to iron vacancies in this material, even though iron vacancies remain the more stable point defect under these conditions.

### Iron Vacancies for Octahedral Sites in Magnetite

We begin our discussion by focusing on iron vacancies on octahedral sites only. We also calculated the formation energies of iron vacancies at tetrahedral sites and found them higher in energy than the octahedral ones by about a 1 eV. Similar to our previous work on oxygen vacancies,^32^ the iron vacancies on the octahedral sublattice in the low-temperature monoclinic phase can be classified according to the relative number of Fe^3+^ and Fe^2+^ in the next-nearest-neighbor shell, the nearest ions being O^2–^. The choice of this criterion is motivated by the role of Fe^3+^ and Fe^2+^ as electron and hole localization sites, respectively.

### Local Environments for Iron Vacancies in Magnetite

Each iron site in the low-temperature monoclinic phase structure has six Fe ions in the neighborhood, each of which can be occupied by Fe^3+^ or Fe^2+^. In the 224-atom monoclinic cell, there are eight different Wyckoff sites for octahedral Fe^2+^ and Fe^3+^ each. For both Fe^2+^ and Fe^3+^, each of these eight sites falls into one of the five categories of local environments found around the octahedral Fe sites. Schematics showing different local chemical environments around the Fe^2+^ and Fe^3+^ sites on the octahedral sublattice are shown in Figures 1 and 2.

**Figure 1:**
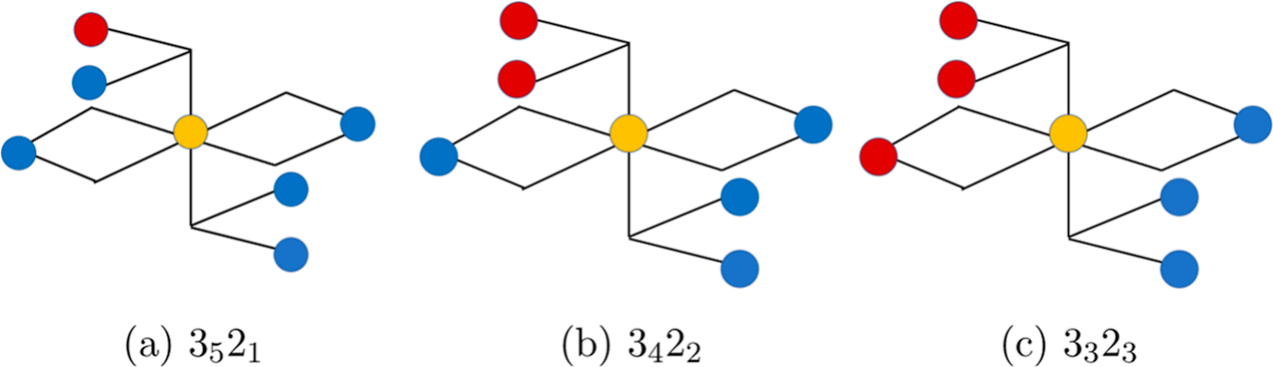
Local environments around Fe^2+^ sites on the octahedral sublattice of the monoclinic phase of magnetite. The red, blue, and yellow circles denote Fe^2+^, Fe^3+^, and the octahedrally coordinated Fe^2+^ vacancy sites, respectively. Subfigures (a), (b), and (c) show environments with 5, 4, and 3 Fe^3+^ neighbors, respectively.

**Figure 2:**
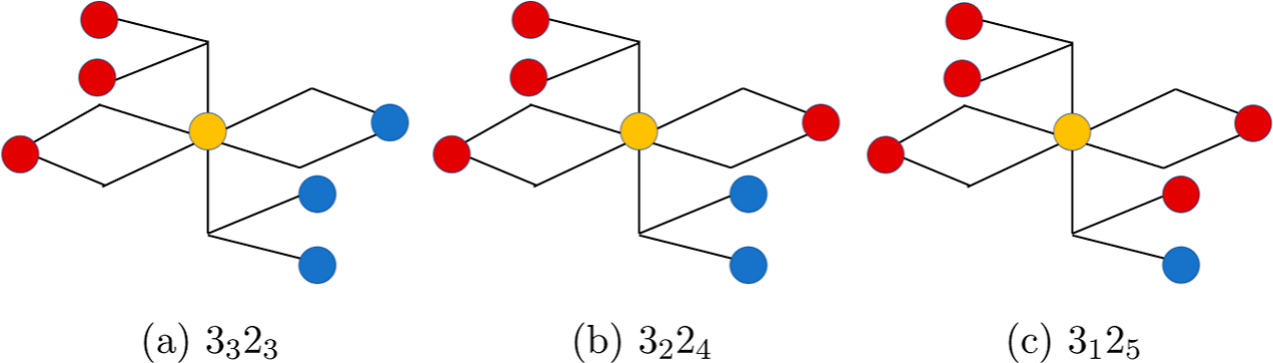
Local environments around Fe^3+^ sites on the octahedral sublattice of the monoclinic phase of magnetite. The red, blue, and yellow circles denote Fe^2+^, Fe^3+^, and the octahedrally coordinated Fe^3+^ vacancy sites, respectively. Subfigures (a), (b), and (c) show environments with 3, 2, and 1 Fe^3+^ neighbors, respectively.

Similar to our previous work on oxygen vacancies,^32^ we describe the charge and spin configuration as well as the local environment of an iron vacancy in magnetite using an extended Kröger–Vink notation. In the octahedral sublattice, each Fe site has six octahedrally coordinated Fe ions in the next-nearest-neighbor shell with a shared O^2–^ ion with each of them (see Figures 1 and 2). We call the vacancy plus the six neighboring Fe ions the defect core. To account for this difference in the local environment compared to oxygen vacancies, we modify our previously used notation for oxygen vacancies to be the following for iron vacancies:

ENV:SPIN:CHARGE

where the environment is specified by the charge on the octahedral Fe cations within the core before the vacancy is introduced. For ENV = 3_*n*_2_6–*n*_, the subscripts denote the number of Fe ions of the Fe^3+^ or Fe^2+^ type in the local environment (see Figures 1 and 2). The spin configurations (SPIN) of the excess electrons/holes are specified as up (U) or down (D), and the charge configuration is indicated using Kröger–Vink notation for the core (V_Fe_^′^, V_Fe_, or V_Fe_^•^) and bound electrons (Fe_Fe_^′^) or holes (Fe_Fe_^•^) localized on Fe cations that are neighbors to, but outside, the core. In this notation, spin labels for electrons are defined relative to the magnetic order in low-temperature magnetite. Magnetite remains ferrimagnetic up to approximately 850 K, with oppositely aligned net moments between Fe ions in the octahedral and tetrahedral sublattices. We designate the octahedral sublattice as “spin-up” and the tetrahedral sublattice as “spin-down.” Spin-polarized DFT calculations for Fe vacancies are performed without constraints, and the spin state of the defect is expressed in terms of the localized holes or electrons introduced by the vacancy. For example, a neutral iron vacancy with four Fe^3+^ and two Fe^2+^ in the core and both localized electrons being “spin-down” will be named as 3_4_2_2_:DD:0.

### Formation Energies of Octahedral Iron Vacancies

We performed charged defect calculations for iron vacancies with charge states ranging from −3 to 0 for all 16 Wyckoff positions for octahedrally coordinated Fe^2+^ and Fe^3+^ sites. For each charge state for each configuration, the defect configuration consists of an iron vacancy, localized holes in the next nearest-neighbor shell (“Fe vacancy core”), and localized holes outside the core. The Fe vacancy core can be neutral or negatively charged.

The formation energy values for different sites with the same charge ened in Table 1 (for the Fermi level at the valence band maximum (VBM)) and plotted as a function of the Fermi level referenced to the valence band maximum in Figure 3. As shown in Figure 3, octahedrally coordinated iron vacancies can exist in charge states ranging from −3 to neutral. At Fermi levels near the valence band maximum, neutral charge states are preferred. We found no discernible trend in how formation energies vary with the local environment, likely because of similar charge localization across all environments for each charge state. In environments with multiple Wyckoff positions, the formation energies for neutral iron vacancies range from 0.1 to 0.28 eV. Variations in formation energies within the same local environment can be attributed to a difference in charge localization. For example, in the 3_2_2_4_ environment, four different Wyckoff sites require redistribution of three holes in the neutral configuration, yielding formation energies of 1.87, 1.89, 2.00, and 2.08 eV. In the first two cases, all localized holes occupy different octahedral planes, minimizing like-charge repulsion, which may explain the slightly lower formation energy values.

**Figure 3:**
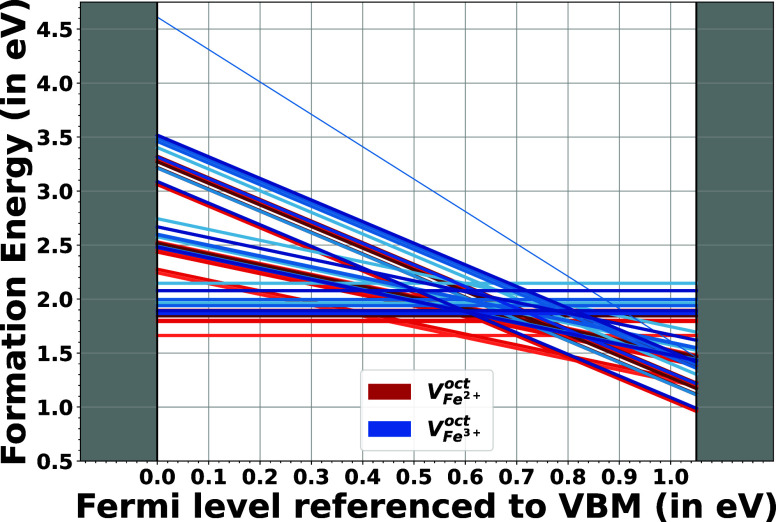
Formation energy of iron vacancies in magnetite for 16 different Wyckoff sites and three charge states: 0, −1, −2, and −3. The red lines depict the vacancies at Fe^2+^ sites, and the blue lines represent the vacancies at the Fe^3+^ sites, with different color gradients indicating different Wyckoff sites. The −3 charge state is only depicted for the 3_5_2_1_ environment, as the rest are not stable with respect to decomposition to −2 charged vacancies and an unbound electron polaron.

### Charge Redistribution around Iron Vacancies

The formation of iron vacancies in magnetite is accompanied by the redistribution of charges around the vacancy core. Charge redistribution around the iron vacancies can be described in terms of localized holes on the octahedral sublattice. Removing a cation can result in neutral or negatively charged vacancies. In the case of magnetite, Fe exists in the form of Fe^3+^ or Fe^2+^. Creating a neutral vacancy will require redistributing either a +3 or +2 positive charge (in the form of localized holes) around the vacancy site. This is in contrast to neutral oxygen vacancies, where the neutral vacancy is accompanied by a redistribution of electrons around the vacancy site. As a result, charge redistribution in the case of Fe vacancies will require the presence of Fe^2+^ ions, which can act as a site for localizing holes by oxidizing to Fe^3+^. As described in Figures 1 and 2, all octahedrally coordinated Fe^3+^ sites have at least three Fe^2+^ atoms in the first nearest-neighbor shell, so there are three available sites for hole localization close to the vacancy site. In the case of Fe^2+^ sites, as shown in Figure 1, the three possible environments are 3_3_2_3_, 3_4_2_2_, and 3_5_2_1_ with three, two, and one Fe^2+^ sites in the core, respectively. Therefore, for neutral and −1 charge states, excess holes need to be localized outside the core for some of these environments.

The core charges and charges outside the core are described in Tables 2 and 3. In cases where the core charge does not match the overall defect charge, excess electrons or holes localize outside the core region. The fourth column in Tables 2 and 3 reports the binding energy of this excess charge. We note that not all charge states (−3, −2, −1, and 0) are stable for all local environments as excess charge in some cases is not bound to the core, effectively making the defect configuration in that charge state spontaneously dissociate into another charge state and a free excess charge.

**Table 1:** Formation Energies of Iron Vacancies with Neutral, −1, −2, and −3 Charge States Calculated for the Fermi Level at the Valence Band Maxima^a^

site occupancy prior to vacancy formation	iron vacancy configuration	charge configuration	*E*_form_ (eV) for different sites
Fe^2+^	3_3_2_3_:DD:0	*V*_Fe_^X^	1.88, 1.96, 1.86
Fe^2+^	3_3_2_3_:D:–1	*V*_Fe_^′^	2.47, 2.52, 2.51
Fe^2+^	3_3_2_3_::–2	*V*_Fe_^″^	3.29, 3.32, 3.27
Fe^2+^	3_4_2_2_:DD:0	*V*_Fe_^X^	1.66, 1.9, 1.8, 1.84
Fe^2+^	3_4_2_2_:D:–1	*V*_Fe_^′^	2.24, 2.53, 2.44, 2.5
Fe^2+^	3_4_2_2_::–2	*V*_Fe_^″^	3.08, 3.28, 3.21, 3.22
Fe^2+^	3_5_2_1_:DD:0	*V*_Fe_^′^ + h•	1.79
Fe^2+^	3_5_2_1_:D:–1	*V*_Fe_^′^	2.28
Fe^2+^	3_5_2_1_::–2	*V*_Fe_^″^	3.06
Fe^3+^	3_1_2_5_:DDD:0	*V*_Fe_^X^	1.94
Fe^3+^	3_1_2_5_:DD:–1	*V*_Fe_^′^	2.59
Fe^3+^	3_1_2_5_:D:–2	*V*_Fe_^″^	3.46
Fe^3+^	3_1_2_5_::–3	*V*_Fe_^‴^	4.61
Fe^3+^	3_2_2_4_:DDD:0	*V*_Fe_^X^	1.87, 2.0, 1.89, 2.08
Fe^3+^	3_2_2_4_:DD:–1	*V*_Fe_^′^	2.49, 2.6, 2.48, 2.67
Fe^3+^	3_2_2_4_:D:–2	*V*_Fe_^″^	N/A, 3.49, N/A, 3.52
Fe^3+^	3_3_2_3_:DDD:0	*V*_Fe_^X^	1.96, 2.15, 1.87
Fe^3+^	3_3_2_3_:DD:–1	*V*_Fe_^′^	2.58, 2.74, 2.48
Fe^3+^	3_3_2_3_:D:–2	*V*_Fe_^″^	3.4, 3.51, 3.32

a The first three columns specify the environment, spin, and charge configurations, and the fourth lists the corresponding calculated defect formation energy. The first three rows are the environments around octahedral Fe^2+^ sites, whereas the last three rows denote the local environments around Fe^3+^ sites on the octahedral sublattice.

**Table 2:** Charge Redistribution around Fe^2+^ Vacancy Sites^a^

environment	charge state	core charge	binding energy
3_3_2_3_	0	0	N/A
	–1	–1	N/A
	–2	–2	N/A
	–3	–2	excess e^–^ not bound
3_4_2_2_	0	0	N/A
	–1	–1	N/A
	–2	–2	N/A
	–3	–2	excess e^–^ not bound
3_5_2_1_	0	–1	0.143 eV
	–1	–1	N/A
	–2	–2	N/A
	–3	–2	excess e^–^ not bound

a The first column lists the local environment by the number of Fe^3+^ and Fe^2+^ neighbors (as explained in the main text). The second column shows the defect charge state, the third gives the charge within the core after relaxation, and the fourth column lists the binding energy of any excess charge localized outside the core.

**Table 3:** Charge Redistribution around Fe^3+^ Vacancy Sites^a^

environment	charge state	core charge	binding energy
3_3_2_3_	0	0	N/A
	–1	–1	N/A
	–2	–2	N/A
	–3	–2	excess e^–^ not bound
3_2_2_4_	0	0	N/A
	–1	–1	N/A
	–2	–2	N/A
		–1	excess e^–^ not bound
	–3	–2	excess e^–^ not bound
3_1_2_5_	0	0	N/A
	–1	–1	N/A
	–2	–2	N/A
	–3	–2	excess e^–^ not bound
	–3	–3	N/A

a The first column lists the local environment by the number of Fe^3+^ and Fe^2+^ neighbors (as explained in the main text). The second column shows the defect charge state, the third gives the charge within the core after relaxation, and the fourth column lists the binding energy of any excess charge localized outside the core.

We first discuss the vacancies formed at the Fe^2+^ sites. For neutral vacancies, the two holes typically localize within the vacancy core on two of the six nearest octahedral Fe sites. In the 3_5_2_1_ environment, where only one Fe^2+^ site is available, one hole localizes outside the core and is weakly bound, with a binding energy of ∼0.14 eV. For the −1 and −2 charge states, the charge remains within the core, containing one and zero holes, respectively. In all −3 charge state configurations, the core charge is −2, with the additional electron remaining unbound, rendering the −3 charge state unstable.

For vacancies located at Fe^3+^ sites, the neutral and −1 charge states retain all charge within the core. In the −2 charge state, two of the 3_2_2_4_ configurations relax to a −2 charged core, while the remaining two configurations maintain a −1 core with one unbound electron. For the −3 charge state, all but one configuration relaxes to a −2 core with an unbound electron; the remaining case forms a −3 charged core but is significantly higher in energy and is unlikely to occur in appreciable concentration under equilibrium conditions near the valence band maximum.

Overall, in iron vacancies in magnetite (Fe_3_O_4_), excess charge beyond −2 typically leads to instability of the charge state with the excess electron localized outside the core and unbound. For all environments except 3_1_2_5_, the most negative stable charge state is −2.

### Iron–Oxygen Divacancies in Magnetite (Fe_3_O_4_)

We next focused on investigating Fe–O divacancies in iron oxides, beginning with magnetite. The chemical potentials used to calculate formation energies for iron vacancies and Fe–O divacancies correspond to oxidizing conditions characterized by maintaining an equilibrium between magnetite (Fe_3_O_4_) and hematite (Fe_2_O_3_).

### Local Chemical Environments for Iron–Oxygen Divacancies in Magnetite (Fe_3_O_4_)

As hypothesized in tracer diffusion studies,^30^ the defect responsible for oxygen transport changes from oxygen vacancy in oxygen-poor conditions to Fe–O divacancy in oxygen-rich environments. Motivated by this hypothesis and the four possible oxygen vacancy environments that were studied in our previous work,^32^ we classify four different classes of divacancy environments with each of them having four different divacancy configurations, depending on which of the four iron sites in the vacancy core is part of the oxygen vacancy–iron vacancy complex. Three of these Fe–O divacancies involve the iron vacancy in the octahedral sublattice, whereas the other involves the iron ions in the tetrahedral sites. The different possible configurations are schematically depicted in Figure 4. The “divacancy core” is defined as the iron and oxygen vacancy sites plus all of the nearest iron neighbors for the oxygen and iron site involved in the divacancy.

**Figure 4:**
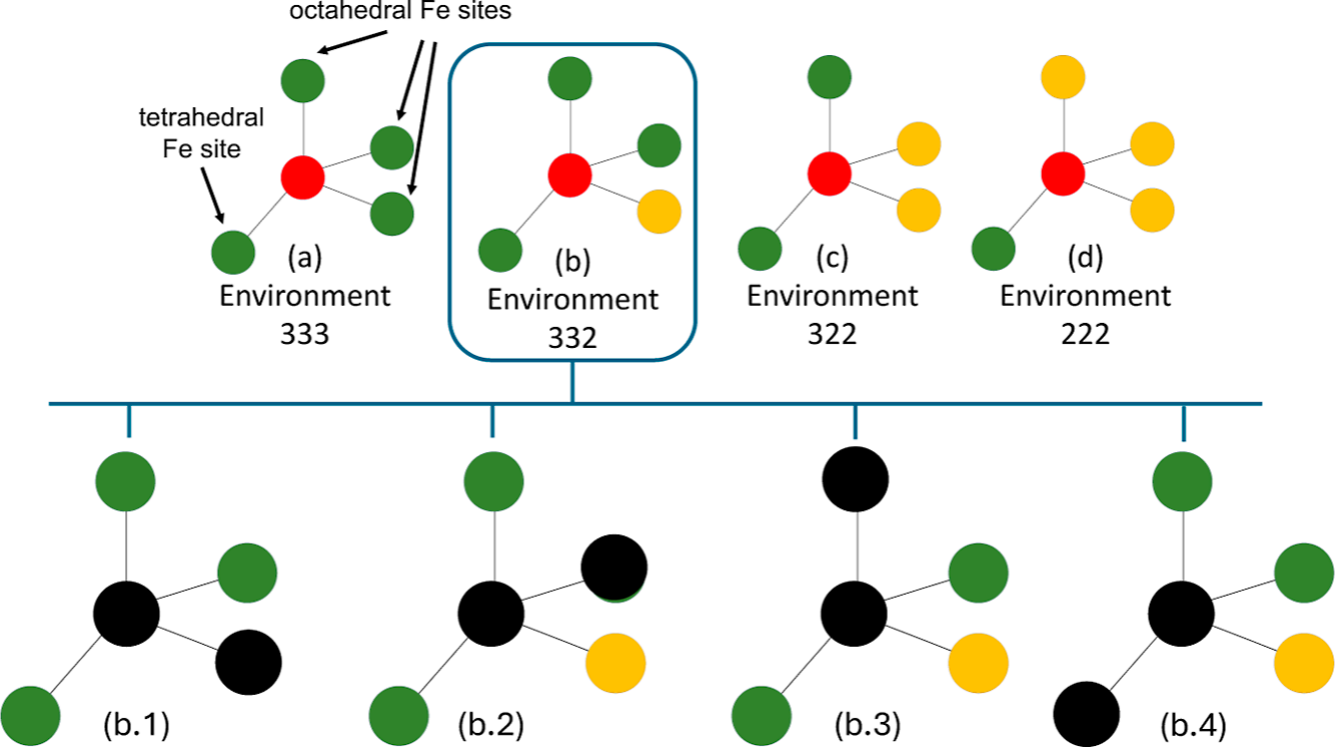
Different configurations for iron–oxygen divacancies in magnetite. The top row shows the four environments of an oxygen site, following the representation introduced in our previous work.^32^ Subfigures (a), (b), (c), and (d) correspond to the 333, 332, 322, and 222 environments, containing three, two, one, and zero octahedral Fe^3+^ sites in the divacancy core, respectively. Subfigures (b.1), (b.2), (b.3), and (b.4) illustrate four distinct Fe–O divacancy configurations within the 332 environment, with black spheres indicating the divacancy sites. The red, green, and yellow spheres denote sites occupied by O^2–^, Fe^3+^, and Fe^2+^, respectively, in the bulk structure. Adapted from ref 32. Available under a CC-BY 4.0 license. Copyright 2023 Shivani Srivastava, Blas Pedro Uberuaga, and Mark Asta.

### Formation Energies of Iron–Oxygen Divacancies

The formation energy of the divacancy associated with a specific oxygen vacancy site depends on the iron vacancy site bound to the oxygen vacancy. Since each oxygen is coordinated with three octahedral and one tetrahedral iron, there are four divacancy configurations for each divacancy site. Table 4 presents the calculated divacancy formation energies for each combination of the four oxygen vacancy environments and four iron vacancy sites (16 in total), with the Fermi level at the valence band edge. These results are also plotted as a function of the Fermi level for each oxygen local environment in Figure 5. As observed, for all four local environments of the oxygen vacancy, the divacancy involving a vacancy at the tetrahedral Fe site exhibits a higher formation energy compared with the one involving the octahedral Fe site at the same oxygen vacancy.

**Table 4:** Formation Energies of Neutral and +1 Charged Fe–O Divacancies^a^

oxygen vacancy environment	iron site valence	*E*_form_ (eV) [neutral]	*E*_form_ (eV) [+1]
333	+3	2.15	1.72
	+3	2.36	1.98
	+3	2.09	1.85
	+3 (tet)	2.75	2.44
332	+3	2.02	1.66
	+3	2.19	1.74
	+2	2.00	1.66
	+3 (tet)	3.05	2.30
322	+3	2.08	1.59
	+2	2.24	1.76
	+2	1.86	1.55
	+3 (tet)	2.82	2.45
222	+3	1.91	1.58
	+2	1.95	1.57
	+2	2.03	1.70
	+3 (tet)	2.85	2.36

a The first two columns specify the oxygen environment and charge configurations of the Fe cation site, respectively. The third and fourth columns list the corresponding calculated defect formation energy for the neutral and +1 charged divacancy configurations, with the value of the Fermi energy corresponding to the valence band edge.

**Figure 5:**
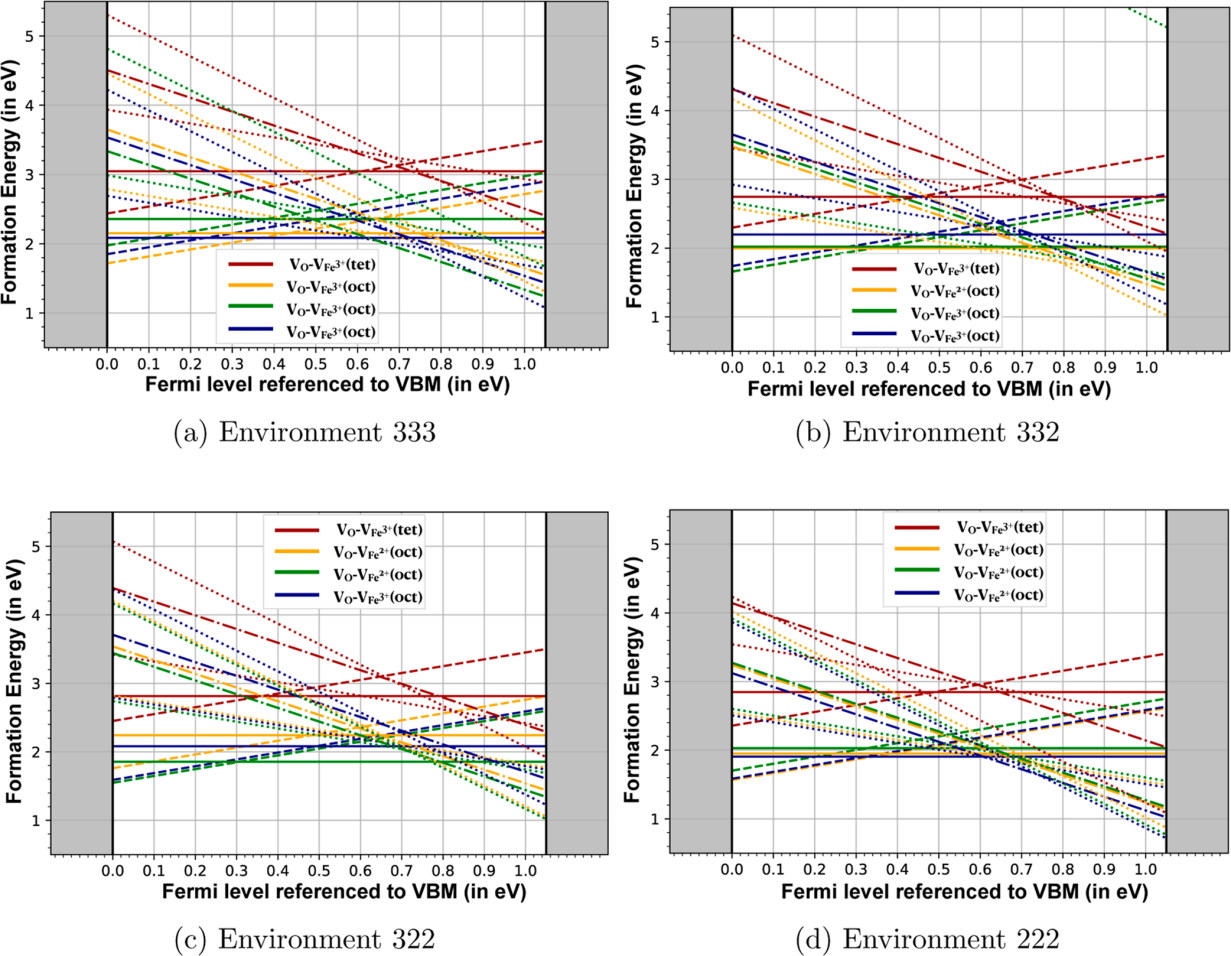
Formation energies for different charge states of Fe–O divacancies formed at the four oxygen sites in magnetite. Legends in each plot show the iron and oxygen vacancy sites for the divacancy in the 448-atom monoclinic supercell. Subfigures (a), (b), (c), and (d) correspond to the 333, 332, 322, and 222 environments, containing three, two, one, and zero octahedral Fe^3+^ sites in the divacancy core, respectively.

### Charge Redistribution around Iron–Oxygen Divacancies

We decompose the formation energy of divacancies in terms of charge states to see whether there are any preferred environments for a specific charge state. As can be seen in Figure 6, the formation of divacancy involving the 222 oxygen vacancy site becomes more stable as the overall charge state of the divacancy becomes more negative, changing from 223 for +1 charged divacancy to 222 for −2 charged divacancy.

**Figure 6:**
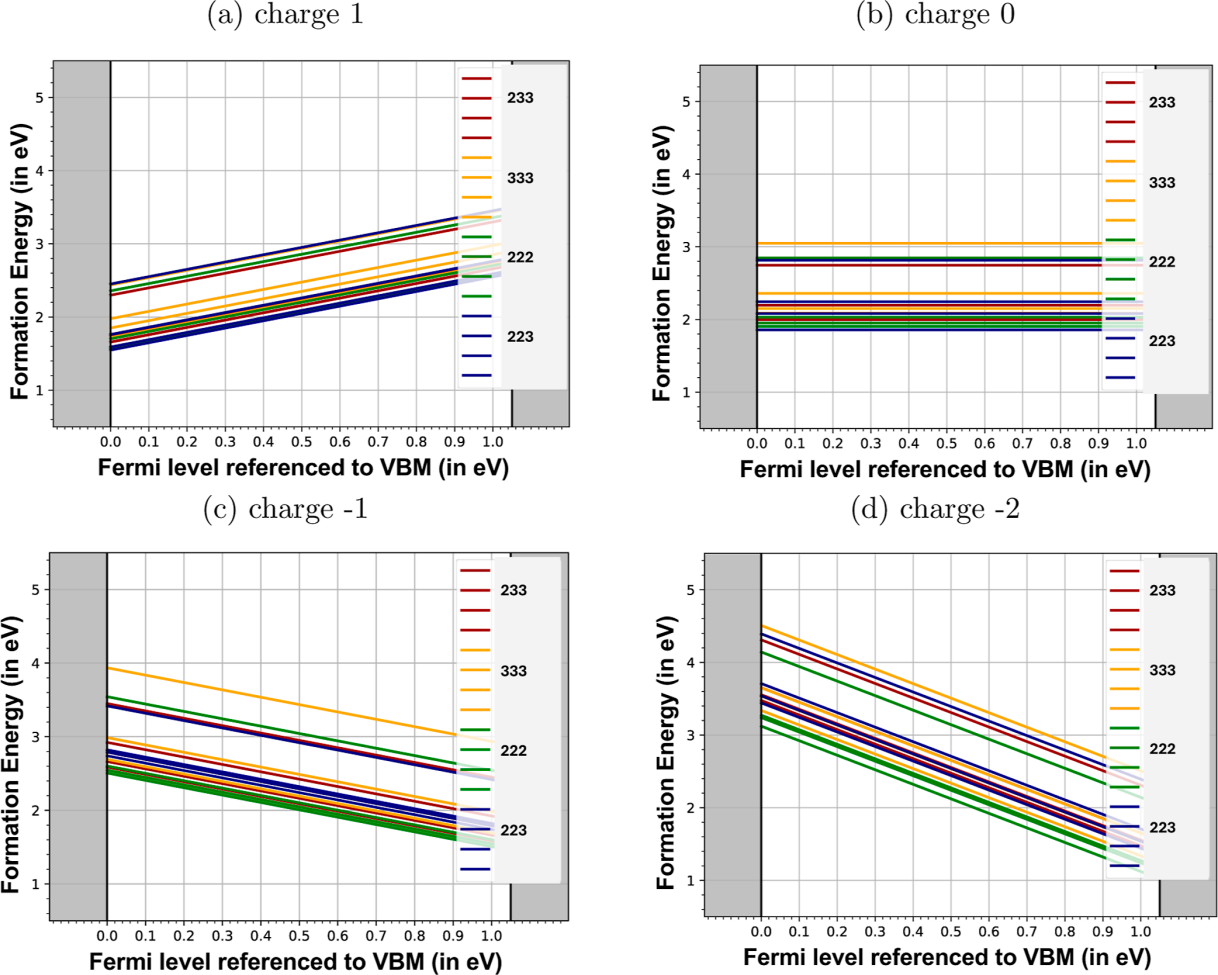
Formation energies of Fe–O divacancies formed at the four different oxygen sites in magnetite for charge states +1, 0, −1, and −2, as shown in subfigures (a), (b), (c), and (d), respectively. The colors for the lines correspond to different environments for the oxygen and iron vacancies as described in the text and legend. The −3 charged divacancies are not stable except for a single configuration as described in the text.

We analyze the charge redistribution around the divacancy by identifying sites of localized holes and electrons and their positions relative to the divacancy site. Depending on the charge state of the iron vacancy site in the divacancy, the amount of charge that needs to be redistributed around the divacancy changes. For example, creating a neutral divacancy for an O^2–^–Fe^2+^ pair does not require creating excess electrons or holes, whereas creating a neutral divacancy for an O^2–^–Fe^3+^ pair will require localizing an excess negative charge in the defect supercell, either by removing a hole with respect to the +1 charged configuration or by localizing an electron on a neighboring Fe^3+^ site. This Fe^3+^ site can be either inside or outside the “divacancy core” (explained below) and can be bound or unbound to the “divacancy core”.

The “divacancy core” consists of the iron and oxygen vacancy sites along with their closest iron neighbors. Depending on the charge state and the local environment of the oxygen vacancy, charge redistribution may affect only the iron sites within the core or extend to iron ions outside it. The core charges for different divacancy sites are shown in Figure 7, followed by a discussion of the binding energy of localized holes and electrons for cases where the excess charge (the difference between the defect charge state and core charge) is nonzero.

**Figure 7:**
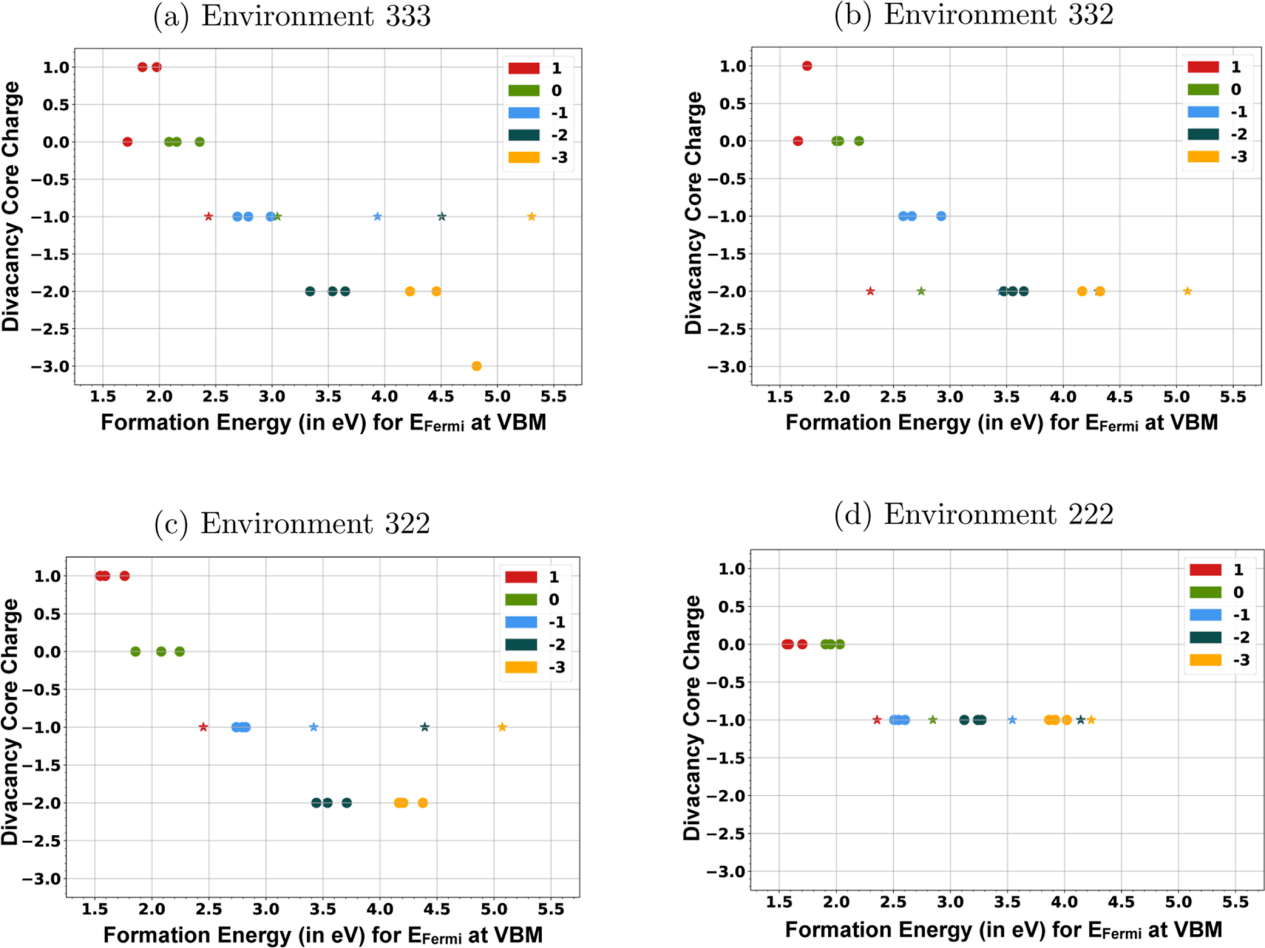
Core charges and formation energies for different defect charge states across divacancy local environments, classified by the oxygen site involved. Points marked by “o” and “*” denote divacancies with iron vacancies on octahedral and tetrahedral sublattices, respectively. Formation energies are shown for the Fermi level at the valence band maximum (VBM), with symbol colors corresponding to defect charge states. Subfigures (a), (b), (c), and (d) correspond to the 333, 332, 322, and 222 environments, containing three, two, one, and zero octahedral Fe^3+^ sites in the divacancy core, respectively.

As can be seen in Figure 7, the core charges of distinct divacancy sites vary differently with the overall charge state of the defect. The excess charges outside the core can be bound or unbound to the core. For the case where the charge outside the core is an electron, the binding energy to the core is calculated by taking a difference between the divacancy formation energy with the sum of the formation energy of the defect with a single electron localized on the lowest-energy Fe^3+^ site and the formation of the divacancy at the same Fe–O vacancy site but a higher charge state with the same core charge. Similarly, for the defect configurations with excess holes localized outside the core, the binding energy of the out-of-core hole is calculated by taking the difference of divacancy formation energy with the sum of formation energy of the hole localized on the most stable Fe^2+^ site and the divacancy with the same Fe–O vacancy sites but a lower charge state with the same core charge.

We initially focused on divacancies where the iron vacancy exists on the octahedral sublattice. For all cases but one, a −3 charged divacancy has either a −2 or −1 charged core. In all the cases where the core charge is −2 for the −3 charged defect configuration, the excess electron (localized on a previous Fe^3+^ site) is not bound to the core. In our model of magnetite, this will effectively mean that the −3 charge state for divacancy is not stable at these sites and will always decompose into a −2 charged divacancy (if stable) and an excess electron polaron. This holds true in our magnetite calculations, irrespective of whether the octahedral iron site involved in the divacancy is Fe^2+^ or Fe^3+^ in the bulk.

The divacancies with the 222 oxygen environment exhibit a different trend, where all of the negatively charged defect states have a −1 charged core. The −1 charged divacancy consists of the Fe and O ions removed plus an electron localized on the tetrahedral site closest to the oxygen vacancy site. This core charge distribution is maintained, and the −2 and −3 divacancy configurations consist of one and two excess electrons localized on Fe^3+^ sites, respectively. In both of these cases, either the excess electrons outside the core are unbound or have very small binding energy (∼5 meV).

As can be seen from the plots in Figure 7, for all of the environments, the neutral and −1 charged divacancies have their complete charge localized inside the “divacancy core”. However, in the case of +1 charged divacancies (denoted by red color), both +1 charged and neutral cores are stable. Depending on the local environment around the oxygen vacancy sites, for a +1 charged divacancy, we see the following configurations. For the 333 and 233 oxygen vacancy environments, the excess hole is strongly bound by ∼100 meV and ∼120 meV, respectively. For all of the 223 environments involved, the cores are +1 charged with no excess holes. For the 222 oxygen vacancy environment, the excess hole is either very weakly bound to the core (∼40 meV) or unbound.

The divacancies involving a tetrahedral iron vacancy (denoted by * in Figure 7) exhibit an interesting trend in divacancy core charge for all four classes of divacancies. For these divacancies, different defect configurations maintain their core charge across changing charge states. The 332 environment is stable with a −2 core charge, whereas the rest of the environments prefer to maintain a −1 charged core.

Summarizing the results for formation energy and charge redistribution of Fe–O divacancies, we find that their stability and charge localization strongly depend on the local environment and defect charge state. As the divacancy charge becomes more negative, configurations involving the 222 oxygen site become increasingly favorable. Charge redistribution analysis shows that for most neutral and −1 charged defects, charge remains within the divacancy core (defined as the O site in the vacancy pair and its four nearest Fe neighbors), while higher charge states often lead to excess electrons or holes localized outside the core, many of which are unbound, especially in the −3 state. This suggests that some highly charged states are not stable. Divacancies involving tetrahedral Fe sites show consistent core charge behavior across charge states, while divacancies involving octahedral Fe sites exhibit larger variation, depending on the local environment.

### Binding Energies of Divacancies in Magnetite

In the absence of any external doping or externally biased Fermi level, magnetite is known to exist as a p-type semiconductor. Hence, we will restrict ourselves to Fermi levels close to the valence band maximum (VBM) while discussing binding energies of the divacancies. Assuming the Fermi level is within 0.3 eV of the valence band maxima, as described in the previously published study,^32^ all the oxygen vacancy sites except the ones in the 333 environment are most stable in the +1 state. For the oxygen vacancy in the 333 environment, the +2 charge state is the most stable. In this range of the Fermi level, all iron vacancies are neutral. Considering only the most stable charge states for the Fermi level at the valence band maximum, we can calculate the binding energy of Fe–O divacancies using the defect reactions:

2 $$V_{Fe-O}^{\cdot }>V_{Fe}^{X}+V_{O}^{\cdot }$$

for all oxygen sites except 333, where

3 $$V_{Fe-O}^{\cdot }>V_{Fe}^{X}+V_{O}^{\cdot \cdot }+e^{-}$$

According to this definition, a positive binding energy describes a favorable tendency for oxygen and iron vacancies to form a stable complex. As can be seen from the binding energy values listed in Table 5, the iron and oxygen divacancies in magnetite are strongly bound.

**Table 5:** Binding Energies of +1 Charged Fe–O Divacancies for the Fermi Level at the Valence Band Maxima^a^

environment	iron site charge	*E*_binding_ (eV)
333	+3	0.22
	+3	0.44
	+3	0.28
332	+3	0.43
	+3	0.77
	+2	0.67
322	+3	0.99
	+2	0.54
	+2	0.82
222	+2	0.8
	+2	0.58
	+2	0.76

a The first two columns specify the oxygen environment and charge state of the Fe site before the vacancy is introduced. The third column lists the corresponding calculated divacancy binding energy as described in the text.

These results in Table 5 along with those in Tables 4 and 1, indicating that iron–oxygen divacancies have lower formation energies than isolated iron vacancies under oxidizing conditions and exhibit a strong binding tendency, support the prior hypothesis that such divacancies could play a critical role in oxygen transport in magnetite under oxidizing conditions.^30^

We end this section by summarizing the key takeaways from the results of Fe–O divacancies in magnetite. Our calculations show that for certain divacancy configurations in magnetite, the +1 charge state exhibits formation energies comparable to those of neutral iron vacancies under oxidizing conditions. The calculated binding energies for divacancy complexes in magnetite studied in this work lie between 0.2 and 1.0 eV. These values are significantly larger than the thermal energy at room temperature (*kT* ∼ 0.025 eV), suggesting that once formed, these complexes are unlikely to dissociate spontaneously at moderate temperatures. As discussed in previous works,^53^ for the concentration of complexes to be comparable to isolated defects when created under thermal equilibrium, the binding energies as defined here should not only be positive but should also be comparable in magnitude with the formation energy of isolated defects. However, when formed under nonequilibrium conditions such as under irradiation, divacancies may initially form in high concentrations and, if their binding energies are sufficiently large, remain kinetically stable over extended periods. We therefore use the term “strongly bound” for these divacancies, emphasizing the kinetic rather than purely thermodynamic stability of the complexes. In the case of magnetite, these results therefore point to the possibility that iron diffusion, assumed to be mediated by iron vacancies, would be impacted by the formation of complexes with oxygen vacancies under oxidizing conditions,^30,31^ especially when exposed to irradiation conditions.

### Iron–Oxygen Divacancy in Fe_2_O_3_ (Hematite)

We end the discussion on defect complexes by briefly looking at a different iron oxide: Fe_2_O_3_ (hematite). Unlike magnetite (Fe_3_O_4_), hematite (Fe_2_O_3_) contains Fe cations only in the 3+ oxidation state. Therefore, undoped hematite tends to be n-type at finite temperature due to the presence of stable electron polarons formed by reduction of Fe^3+^ ions to Fe^2+^.^55^ A significant body of research has focused on point defects, such as vacancies and interstitials, in hematite. To further understand point defects formed when oxidized iron films are irradiated, we performed additional calculations on cation–anion divacancies and their binding energies in hematite, as both magnetite and hematite can be part of the multiphase scale during iron oxidation.^55^ These findings extend earlier studies.^42,56^ The computational approach, including DFT parameters, supercell sizes, and convergence criteria, follows that in the previous work,^42^ with consistent values for iron and oxygen chemical potentials. We compare the formation energies of divacancies with those of iron and oxygen vacancies and calculate their binding energies relative to their decomposition into individual vacancies and free electrons. Formation energies of different charge states are shown in Figure 8, with iron and oxygen vacancy values taken from the referenced works.^42,56^ Our focus of this study is on divacancy DFT calculations. Under the choice of parameters described in the previous work,^42,56^ the formation energy of *V*_Fe_^–3^ becomes negative above ∼1.4 eV, which may arise from a combination of factors, including the choice of chemical potentials and limitations of the exchange–correlation functional, as well as pinning of Fermi levels due to self-compensation by defects as the Fermi level approaches these values.^57^ To maintain consistency, we used the same *U* values. As our primary focus is on relative energetics and polaron binding, we expect the overall trends and conclusions will still be applicable. When comparing these results to experimental observations, care must be taken to ensure commensurate values of chemical potentials between experiments and simulations.

**Figure 8:**
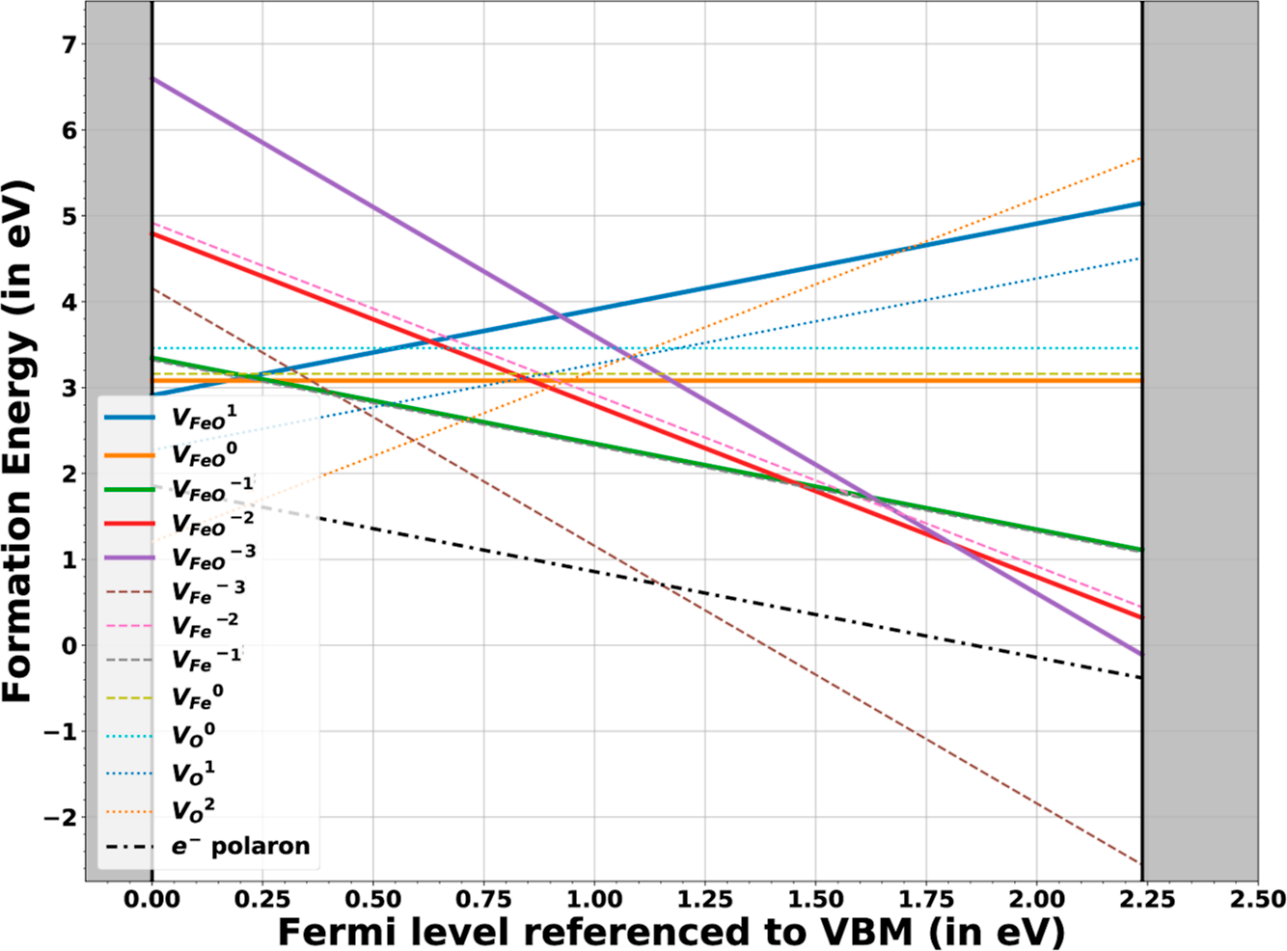
Formation energies of Fe–O divacancies, iron vacancies, and oxygen vacancies in Fe_2_O_3_ (hematite). The solid lines, dashed lines, and dotted lines denote the formation energies of the divacancy, iron vacancy, and oxygen vacancy, respectively.

As can be seen in Figure 8, the dominant defect for Fermi levels above the midgap region (n-type) is the −3 charged iron vacancies. For Fermi levels higher than 0.75 eV above the valence band maxima (VBM), the second most stable vacancy is the Fe–O divacancy, with the most stable vacancy being the −3 charged iron vacancy. The formation energies of all oxygen vacancies are higher for these values at the Fermi level. The most stable charge state of the divacancy changes from +1 to −3 across the bandgap. For Fermi levels above the midgap, the divacancies can form in multiple charge states, transitioning from −1 to −3. The binding energy of the divacancies along with the associated defect reactions are shown in Table 6. As in the case of magnetite, we see the localization of charges in the form of electron polarons in hematite. Recent studies on polarons in hematite show that hybrid functionals predict the electron polaron to be delocalized over two Fe sites.^58^ It should be noted that *U* corrections can sometimes overlocalize polarons, and the energetic effects of this delocalization over neighboring Fe sites are not included in this work. The degree of localization of electron polarons has a significant effect on the binding energies of Fe–O divacancies in hematite and is discussed in the next section.

**Table 6:** Binding Energies of Fe–O Divacancies for Fermi Levels above the Midgap^a^

charge state	defect reaction	*E*_binding_ (eV)
–1	$V_{Fe-O}^{\prime }+2e^{-}\to V_{Fe}^{\prime \prime \prime }+V_{O}^{X}$	0.56
–2	$V_{Fe-O}^{\prime \prime }+1e^{-}\to V_{Fe}^{\prime \prime \prime }+V_{O}^{X}$	0.97
–3	$V_{Fe-O}^{\prime \prime \prime }\to V_{Fe}^{\prime \prime \prime }+V_{O}^{X}$	1.01

a The first two columns specify the divacancy charge state and the respective defect reaction, where *V*_Fe_^‴^ and *V*_O_^X^ are the most stable configurations for the iron and oxygen vacancies used to calculate the binding energy. The third column shows the binding energy for the reaction in column two.

## Discussion

Here, we discuss briefly how the results presented for Fe vacancies compare to the current literature and the interpretation of these results. In experimental diffusion studies, the dominant mechanism for diffusion of iron at high oxygen chemical potentials is through the diffusion of iron vacancies.^11,12^ The change in the slope of the iron tracer diffusion coefficient curves as a function of oxygen partial pressure indicates a transition in the cation diffusion mechanism from an interstitialcy type in reducing conditions to a vacancy type in oxidizing conditions. Previous computational studies on iron vacancies have either focused on the hopping of iron vacancies in metallic cubic spinel and relative formation energies of tetrahedral and octahedral sites (with no charge ordering on the octahedral sublattice)^19^ or considered only the neutral charge state of iron vacancies.^21,22^ We limit our comparison to a qualitative discussion, as variations in supercell sizes, charge ordering on the octahedral sublattice, *U* values, and iron chemical potentials across studies prevent quantitative analysis.

In the studies mentioned above, similar to our results, it is found that the iron vacancies at the tetrahedral sites have much higher formation energy than the ones on the octahedral sublattice. For iron vacancy calculations in the half-metallic inverse spinel structure,^18^ it is found that the formation of iron vacancies is accompanied by a change in the net magnetic moment of the supercell due to the redistribution of holes. However, as the electronic structure of the bulk cell used in these previous studies is half-metallic, this excess charge is delocalized over all of the octahedral sites in the defect supercell. Including the energetics of vacancy formation, which can capture the associated redox of ions, is crucial to model not just iron transport but also the formation of phases such as maghemite during the oxidation of magnetite, which is a result of the ordering of iron vacancies on the magnetite cation sublattice. The results presented in this work would be useful in interpreting the interplay between vacancy formation and changes in the redox state of iron ions in these complex oxide systems.

Divacancies consisting of cation and anion vacancies bound to each other have been hypothesized in several experimental^27,59,60^ and theoretical studies^26,29^ but are relatively less studied computationally. Specifically, in the case of magnetite, the presence of cation–anion divacancies^30^ has been speculated. At higher oxygen partial pressures, where the formation of oxygen vacancies is less favorable, Fe–O divacancies have been hypothesized to be the dominant defect contributing to oxygen transport in magnetite. The presence of these divacancies is also mentioned in other iron-based spinels in the context of the influence of oxygen vacancies on cation transport.^26^ Compared to the binding energy of ∼3.25 eV for *V*_O_–*V*_Al_ in MgAl_2_O_4_, the binding energy of *V*_Fe_–*V*_O_ divacancies in magnetite is smaller in magnitude, indicating that the divacancy complexes in magnetite are weakly bound compared to MgAl_2_O_4_. However, their binding energy (in the range of ∼0.2–1.0 eV) is still much higher than kT at room temperature (∼0.025 eV), implying that once formed, these clusters will not spontaneously dissociate into isolated vacancies. Under extreme environmental conditions such as irradiation, point defects are initially formed in nonequilibrium conditions, which then attempt to approach equilibrium thermodynamic concentrations with processes of diffusion and annihilation by other point and planar defects as well as inducing disorder on cation sublattices.^24^ Though simpler point defects such as monovacancies and interstitials are usually considered to play a dominant role in mass transport, the formation of these defect complexes can be an important factor in determining the overall transport rates and mechanisms. Especially under irradiation, the formation of defect complexes is not uncommon and has been extensively studied in some materials with applications in extreme environments.^24,25,61,62^ Based on the results in previous sections, we can conclude that there is a strong energetic driving force for oxygen and iron vacancies to form clusters in both magnetite (Fe_3_O_4_) and hematite (Fe_2_O_3_). For Fermi energies close to the VBM, the formation energies of divacancies, stable in the +1 charge state, are comparable to those of the neutral iron vacancies in magnetite (Fe_3_O_4_). As the greater number of possible configurations for isolated vacancies compared to divacancies leads to a higher configurational entropy,^53^ the formation of isolated defects is favored at elevated temperatures, even when their formation energies are of similar magnitude. However, divacancy complexes can still persist at lower temperatures if their initial concentrations are high, particularly under nonequilibrium conditions such as irradiation. A similar phenomenon has been observed in the formation of Mg–H complexes in GaN,^53^ where “frozen-in” concentrations from higher temperatures remain significant above equilibrium levels upon cooling.

For all of the local environments considered, the divacancies involving iron vacancies in the tetrahedral site are higher in energy than those with an iron vacancy on the octahedral sublattice. The formation energy of the divacancy exhibits site-dependent variation within each type of environment, with values ranging from 0.08 to 0.38 eV as the iron vacancy occupies different octahedral sites in a local chemical environment. In previous work done on diffusion of cation–anion divacancy in MgAl_2_O_4_,^26^ it was found that rotation is required for these divacancies to diffuse in the spinel lattice. Assuming similar barriers, this will make divacancy reorientation at some sites much more likely than that at others, and the relative stability of different orientations of divacancies can play an important role in determining the principal diffusion pathways. The same study also found that the divacancies involving vacancies in the tetrahedral site are kinetically unstable and relaxed to a configuration with cation vacancies on the octahedral sublattice. Both divacancy types exhibited reduced mobility compared to monovacancies. If a similar behavior holds in magnetite, divacancy formation could significantly influence defect transport under irradiation. While MgAl_2_O_4_ diffusion was modeled using empirical potentials and TAD simulations to access long time scales, such studies remain challenging for magnetite due to the lack of force fields that capture redox and complex defect interaction, which could be a key direction for future work.

Similar to magnetite (Fe_3_O_4_), the results in Table 6 indicate a significant energetic driving force for the oxygen vacancies to bind to the iron vacancies in hematite (Fe_2_O_3_). Iron vacancies still remain the dominant vacancy-type defect for Fermi levels above the midgap, as their formation energy is lower than that of the divacancy (around 2.44 eV for Fermi levels close to the conduction band minima (CBM)). The binding energies of Fe–O divacancies in hematite range from 0.5 to 1.0 eV. Similar to the behavior discussed above for magnetite, under irradiation, nonequilibrium concentrations of vacancies may form in hematite. The stability of Fe–O divacancies can influence the material’s relaxation back to equilibrium. If these complexes are less mobile than isolated iron vacancies, as seen in MgAl_2_O_4_, they may hinder vacancy-driven relaxation mechanisms, following irradiation.

We also note that including the thermodynamics of electron polarons is important for modeling the divacancy binding energy, at least in the low-temperature regimes, where the number of free carriers is limited. The band gap of hematite is about 2.2 eV.^63^ Therefore, for temperatures below 873 K,^63^ hematite shows polaronic conduction. At these temperatures, electron polarons are known to exist and mediate charge transport in hematite.^39,54^ When calculating the binding energy of the divacancies with respect to decomposition into iron and oxygen vacancies, if the electronic charge is involved in the defect reaction, the value of binding energy depends on whether the negative charge is going to a band-like state or a localized polaronic state. For example, for the defect reaction $V_{Fe-O}^{\prime }+2e^{-}\to V_{Fe}^{\prime \prime \prime }+V_{O}^{X}$ , the binding energy of divacancies is 0.56 eV when the formation energy of e^–^ corresponds to electron polaron and around 4.27 eV when the e^–^ corresponds to the charge exchanged with the electron reference potential (VBM in our convention). This enormous difference comes from the localization energy of the electron polaron. At room temperature (∼300 K), where the intrinsic free charge carrier concentration is low, the former should provide a better estimate of the binding energies of divacancies. As mentioned in the previous section, hybrid density functional theories predict an even lower energy configuration of electron polarons in hematite,^39^ which will likely increase the predicted stability of Fe–O divacancies in hematite with respect to decomposition into isolated Fe and O vacancies. At higher temperatures, other entropic terms might play a much more dominant role in determining the binding free energy of these defect complexes. Correctly capturing binding energies is essential for reaction–diffusion-based modeling,^31,64^ often used to simulate the irradiation effect at the mesoscale. Recent experimental studies have explored the effect of irradiation on iron oxide films,^31,55^ and we hope that these results will motivate a critical assessment of the role of Fe–O vacancy complexes in studying complex phenomena such as the effect of irradiation on oxide film growth and transport through these films at temperatures below 873 K.

## Conclusions

Employing DFT + *U* calculations, we analyzed formation energies and charge redistribution associated with iron vacancies and Fe–O divacancies in magnetite (Fe_3_O_4_). Additionally, the relative stability of Fe–O divacancies compared to isolated iron and oxygen vacancies was examined in hematite (Fe_2_O_3_). For divacancies, the binding energy, with respect to decomposition into individual iron and oxygen vacancies, was also calculated. The formation of iron vacancies in iron oxides is also accompanied by the redistribution of charges around the defect core, with the charge being localized within the vacancy core in the majority of cases. Furthermore, the results indicate that in the case of configurations with an overall charge of −3 for vacancies at Fe^2+^ sites, the electron outside the core is not bound to the core, resulting in a −2 charged core with an excess electron. In the case of divacancies in magnetite, we find that there is a strong energetic driving force for oxygen and iron vacancies to form clusters. For Fermi energies near the valence band maximum (VBM), divacancies that are stable in the +1 charge state exhibit formation energies comparable to neutral iron vacancies. The formation energies of divacancies with an iron vacancy at the octahedral site vary in ranges of about 0.08 to 0.38 eV depending on the local environment. For all environments considered, the divacancies involving iron vacancies in the tetrahedral site are higher in energy than those with the iron vacancy on the octahedral sublattice. This could have important implications for determining the principal diffusion pathways at some sites, as reorientation for divacancies involving iron vacancies on a tetrahedral site would be more likely than those with iron vacancies on octahedral sites due to the relative stability of different orientations of divacancies around an oxygen site. The analysis of Fe–O divacancies is further extended to hematite (Fe_2_O_3_), where the results indicate a significant energetic driving force for the oxygen vacancies to bind to the iron vacancies, although iron monovacancies are still predicted to be much more favorable. Atomistic modeling of defect-related processes in iron oxides remains a challenge. Alongside developing better descriptions of exchange–correlation, incorporating finite-temperature effects such as configurational and vibrational entropy will be important for accurate defect stability predictions. Development of charge- and spin-aware interatomic potentials, particularly using machine learning,^65^ will be critical for modeling defect kinetics, redox behavior, and interface processes in iron oxides. The evolution of microstructure as well as irradiation-assisted degradation is governed by the mobility and interaction of point defects and their clusters.^24,25,31,61^ Therefore, understanding defect clustering is crucial for predicting material performance under nonequilibrium conditions typical of nuclear reactors and corrosion-prone environments. The findings presented in this work may support future studies of Fe–O divacancies and their impact on oxide film growth and transport under irradiation.

## Supplementary Material



## Notes

CIF data for magnetite and hematite supercells, dielectric constant, and DFT calculation parameters (PDF)

## ∥

Quantum Simulations Group, Materials Science Division, Lawrence Livermore National Laboratory, Livermore, California 94550–5507, United States
