# Supplementary material for: Density Functional Theory Study of Iron–Oxygen Divacancies in Magnetite (Fe3O4) and Hematite (Fe2O3)
Source: J Phys Chem C Nanomater Interfaces. 2025 Aug 19;129(35):15749–62. doi: 10.1021/acs.jpcc.5c02852 (PMC12415821; doi:10.1021/acs.jpcc.5c02852)
Supplement: Supplementary file 1 [file jp5c02852_si_001.pdf]

Density Functional Theory Study of iron-oxygen divacancies in magnetite (Fe_3O_4) and hematite (Fe_2O_3): Supplementary Information

Shivani Srivastava,[*,†,¶] Blas Pedro Uberuaga,[‡] and Mark Asta[†,¶]

†*Department of Materials Science and Engineering, University of California, Berkeley, CA 94720, USA*

‡*Materials Science and Technology Division, Los Alamos National Laboratory, Los Alamos, NM 87544, USA*

¶*Materials Sciences Division, Lawrence Berkeley National Laboratory, Berkeley, CA 94720, USA*

E-mail: shivani_s@berkeley.edu

CIF Files for Magnetite and Hematite Supercells

Magnetite Supercell CIF

```
data_image0

_chemical_formula_structural       Fe96O128

_chemical_formula_sum              "Fe96 O128"

_cell_length_a       12.0041

_cell_length_b       12.0147
```

[0]Current address: Quantum Simulations Group, Materials Science Division, Lawrence Livermore National Laboratory, Livermore, California 94550-5507, United States

```
_cell_length_c        16.9571
_cell_angle_alpha     90.0001
_cell_angle_beta      89.9735
_cell_angle_gamma     89.9999

_space_group_name_H-M_alt    "P 1"
_space_group_IT_number       1

loop_
  _space_group_symop_operation_xyz
  'x, y, z'

loop_
  _atom_site_type_symbol
  _atom_site_label
  _atom_site_symmetry_multiplicity
  _atom_site_fract_x
  _atom_site_fract_y
  _atom_site_fract_z
  _atom_site_occupancy
  Fe  Fe1       1.0  0.87600  0.25089  0.56281  1.0000
  Fe  Fe2       1.0  0.87598  0.74905  0.06281  1.0000
  Fe  Fe3       1.0  0.37599  0.75091  0.56280  1.0000
  Fe  Fe4       1.0  0.37598  0.24904  0.06280  1.0000
  Fe  Fe5       1.0  0.87900  0.74879  0.56330  1.0000
  Fe  Fe6       1.0  0.87901  0.25114  0.06329  1.0000
  Fe  Fe7       1.0  0.37902  0.24880  0.56329  1.0000
```

Fe	Fe8	1.0	0.37901	0.75115	0.06328	1.0000
Fe	Fe9	1.0	0.62844	0.24891	0.94065	1.0000
Fe	Fe10	1.0	0.62844	0.75102	0.44065	1.0000
Fe	Fe11	1.0	0.12845	0.74893	0.94064	1.0000
Fe	Fe12	1.0	0.12845	0.25102	0.44065	1.0000
Fe	Fe13	1.0	0.62517	0.75404	0.93980	1.0000
Fe	Fe14	1.0	0.62518	0.24590	0.43980	1.0000
Fe	Fe15	1.0	0.12517	0.25405	0.93980	1.0000
Fe	Fe16	1.0	0.12518	0.74589	0.43980	1.0000
Fe	Fe17	1.0	0.87659	0.50122	0.68943	1.0000
Fe	Fe18	1.0	0.87659	0.49872	0.18942	1.0000
Fe	Fe19	1.0	0.37659	0.00121	0.68941	1.0000
Fe	Fe20	1.0	0.37658	0.99873	0.18941	1.0000
Fe	Fe21	1.0	0.87920	0.00271	0.68749	1.0000
Fe	Fe22	1.0	0.87919	0.99722	0.18749	1.0000
Fe	Fe23	1.0	0.37920	0.50270	0.68750	1.0000
Fe	Fe24	1.0	0.37920	0.49722	0.18749	1.0000
Fe	Fe25	1.0	0.62300	0.49688	0.81178	1.0000
Fe	Fe26	1.0	0.62301	0.50305	0.31178	1.0000
Fe	Fe27	1.0	0.12302	0.99687	0.81177	1.0000
Fe	Fe28	1.0	0.12301	0.00306	0.31177	1.0000
Fe	Fe29	1.0	0.62624	0.99422	0.81441	1.0000
Fe	Fe30	1.0	0.62625	0.00571	0.31440	1.0000
Fe	Fe31	1.0	0.12623	0.49421	0.81441	1.0000
Fe	Fe32	1.0	0.12623	0.50572	0.31440	1.0000
Fe	Fe33	1.0	0.74883	0.50020	0.50028	1.0000
Fe	Fe34	1.0	0.74882	0.49972	0.00028	1.0000

Fe	Fe35	1.0	0.24882	0.00021	0.50026	1.0000
Fe	Fe36	1.0	0.24882	0.99974	0.00027	1.0000
Fe	Fe37	1.0	0.00088	0.99909	0.99953	1.0000
Fe	Fe38	1.0	0.00088	0.00085	0.49954	1.0000
Fe	Fe39	1.0	0.50089	0.49909	0.99954	1.0000
Fe	Fe40	1.0	0.50089	0.50085	0.49954	1.0000
Fe	Fe41	1.0	0.00136	0.25115	0.25205	1.0000
Fe	Fe42	1.0	0.00136	0.74879	0.75206	1.0000
Fe	Fe43	1.0	0.50136	0.75114	0.25205	1.0000
Fe	Fe44	1.0	0.50136	0.24878	0.75205	1.0000
Fe	Fe45	1.0	0.62852	0.87454	0.62624	1.0000
Fe	Fe46	1.0	0.62852	0.12540	0.12625	1.0000
Fe	Fe47	1.0	0.12854	0.37453	0.62625	1.0000
Fe	Fe48	1.0	0.12853	0.62542	0.12624	1.0000
Fe	Fe49	1.0	0.75001	0.24880	0.75125	1.0000
Fe	Fe50	1.0	0.75001	0.75113	0.25125	1.0000
Fe	Fe51	1.0	0.25002	0.74879	0.75124	1.0000
Fe	Fe52	1.0	0.25001	0.25114	0.25124	1.0000
Fe	Fe53	1.0	0.75272	0.74937	0.75153	1.0000
Fe	Fe54	1.0	0.75274	0.25057	0.25153	1.0000
Fe	Fe55	1.0	0.25272	0.24936	0.75153	1.0000
Fe	Fe56	1.0	0.25273	0.75057	0.25153	1.0000
Fe	Fe57	1.0	0.87581	0.12383	0.87670	1.0000
Fe	Fe58	1.0	0.87580	0.87610	0.37670	1.0000
Fe	Fe59	1.0	0.37579	0.62382	0.87670	1.0000
Fe	Fe60	1.0	0.37579	0.37612	0.37670	1.0000
Fe	Fe61	1.0	0.87537	0.62498	0.87644	1.0000

Fe	Fe62	1.0	0.87539	0.37497	0.37643	1.0000
Fe	Fe63	1.0	0.37538	0.12497	0.87643	1.0000
Fe	Fe64	1.0	0.37539	0.87497	0.37641	1.0000
Fe	Fe65	1.0	0.25716	0.50039	0.50003	1.0000
Fe	Fe66	1.0	0.75716	0.00038	0.50003	1.0000
Fe	Fe67	1.0	0.75716	0.99956	0.00003	1.0000
Fe	Fe68	1.0	0.25716	0.49957	0.00003	1.0000
Fe	Fe69	1.0	0.00157	0.49905	0.00150	1.0000
Fe	Fe70	1.0	0.00157	0.50089	0.50150	1.0000
Fe	Fe71	1.0	0.50157	0.99905	0.00149	1.0000
Fe	Fe72	1.0	0.50157	0.00091	0.50148	1.0000
Fe	Fe73	1.0	0.87283	0.37692	0.87589	1.0000
Fe	Fe74	1.0	0.87284	0.62301	0.37589	1.0000
Fe	Fe75	1.0	0.37285	0.87692	0.87588	1.0000
Fe	Fe76	1.0	0.37284	0.12302	0.37588	1.0000
Fe	Fe77	1.0	0.87670	0.86814	0.87254	1.0000
Fe	Fe78	1.0	0.87671	0.13180	0.37255	1.0000
Fe	Fe79	1.0	0.37669	0.36813	0.87255	1.0000
Fe	Fe80	1.0	0.37670	0.63180	0.37254	1.0000
Fe	Fe81	1.0	0.99983	0.75135	0.25250	1.0000
Fe	Fe82	1.0	0.99983	0.24859	0.75250	1.0000
Fe	Fe83	1.0	0.49984	0.25135	0.25250	1.0000
Fe	Fe84	1.0	0.49983	0.74858	0.75249	1.0000
Fe	Fe85	1.0	0.62763	0.12457	0.62980	1.0000
Fe	Fe86	1.0	0.62762	0.87536	0.12980	1.0000
Fe	Fe87	1.0	0.12762	0.62457	0.62981	1.0000
Fe	Fe88	1.0	0.12763	0.37537	0.12981	1.0000

Fe	Fe89	1.0	0.62887	0.62876	0.62677	1.0000
Fe	Fe90	1.0	0.62888	0.37117	0.12677	1.0000
Fe	Fe91	1.0	0.12889	0.12875	0.62677	1.0000
Fe	Fe92	1.0	0.12887	0.87118	0.12677	1.0000
Fe	Fe93	1.0	0.62726	0.37538	0.62320	1.0000
Fe	Fe94	1.0	0.62726	0.62455	0.12319	1.0000
Fe	Fe95	1.0	0.12726	0.87538	0.62319	1.0000
Fe	Fe96	1.0	0.12725	0.12456	0.12319	1.0000
O	O1	1.0	0.87175	0.12336	0.49659	1.0000
O	O2	1.0	0.87174	0.87658	0.99659	1.0000
O	O3	1.0	0.37175	0.62336	0.49659	1.0000
O	O4	1.0	0.37174	0.37658	0.99659	1.0000
O	O5	1.0	0.88335	0.62096	0.49587	1.0000
O	O6	1.0	0.88335	0.37897	0.99587	1.0000
O	O7	1.0	0.38334	0.12097	0.49586	1.0000
O	O8	1.0	0.38335	0.87897	0.99586	1.0000
O	O9	1.0	0.62659	0.11460	0.00350	1.0000
O	O10	1.0	0.62659	0.88534	0.50349	1.0000
O	O11	1.0	0.12659	0.61461	0.00349	1.0000
O	O12	1.0	0.12660	0.38534	0.50349	1.0000
O	O13	1.0	0.62612	0.62646	0.00481	1.0000
O	O14	1.0	0.62612	0.37347	0.50482	1.0000
O	O15	1.0	0.12610	0.12647	0.00482	1.0000
O	O16	1.0	0.12611	0.87347	0.50482	1.0000
O	O17	1.0	0.87989	0.38335	0.50045	1.0000
O	O18	1.0	0.87988	0.61660	0.00045	1.0000
O	O19	1.0	0.37989	0.88335	0.50043	1.0000

O	O20	1.0	0.37988	0.11659	0.00044	1.0000
O	O21	1.0	0.87077	0.88007	0.50277	1.0000
O	O22	1.0	0.87077	0.11986	0.00277	1.0000
O	O23	1.0	0.37077	0.38008	0.50277	1.0000
O	O24	1.0	0.37078	0.61987	0.00276	1.0000
O	O25	1.0	0.63016	0.37572	0.00758	1.0000
O	O26	1.0	0.63017	0.62421	0.50758	1.0000
O	O27	1.0	0.13016	0.87573	0.00758	1.0000
O	O28	1.0	0.13016	0.12421	0.50757	1.0000
O	O29	1.0	0.62655	0.88463	0.00642	1.0000
O	O30	1.0	0.62655	0.11531	0.50642	1.0000
O	O31	1.0	0.12655	0.38464	0.00642	1.0000
O	O32	1.0	0.12655	0.61531	0.50643	1.0000
O	O33	1.0	0.87804	0.13364	0.75025	1.0000
O	O34	1.0	0.87803	0.86629	0.25026	1.0000
O	O35	1.0	0.37804	0.63363	0.75025	1.0000
O	O36	1.0	0.37804	0.36630	0.25025	1.0000
O	O37	1.0	0.87690	0.62946	0.75409	1.0000
O	O38	1.0	0.87690	0.37048	0.25408	1.0000
O	O39	1.0	0.37690	0.12945	0.75408	1.0000
O	O40	1.0	0.37690	0.87047	0.25407	1.0000
O	O41	1.0	0.62681	0.11998	0.74731	1.0000
O	O42	1.0	0.62681	0.87994	0.24731	1.0000
O	O43	1.0	0.12681	0.61998	0.74732	1.0000
O	O44	1.0	0.12681	0.37995	0.24731	1.0000
O	O45	1.0	0.61933	0.62830	0.74737	1.0000
O	O46	1.0	0.61934	0.37164	0.24737	1.0000

O	047	1.0	0.11934	0.12830	0.74737	1.0000
O	048	1.0	0.11933	0.87164	0.24737	1.0000
O	049	1.0	0.88295	0.37152	0.75496	1.0000
O	050	1.0	0.88296	0.62842	0.25495	1.0000
O	051	1.0	0.38296	0.87151	0.75494	1.0000
O	052	1.0	0.38296	0.12843	0.25495	1.0000
O	053	1.0	0.87594	0.87740	0.75467	1.0000
O	054	1.0	0.87593	0.12253	0.25467	1.0000
O	055	1.0	0.37592	0.37739	0.75467	1.0000
O	056	1.0	0.37593	0.62253	0.25466	1.0000
O	057	1.0	0.62611	0.36817	0.74537	1.0000
O	058	1.0	0.62611	0.63175	0.24536	1.0000
O	059	1.0	0.12612	0.86817	0.74536	1.0000
O	060	1.0	0.12611	0.13175	0.24536	1.0000
O	061	1.0	0.62399	0.86617	0.74740	1.0000
O	062	1.0	0.62400	0.13377	0.24741	1.0000
O	063	1.0	0.12400	0.36617	0.74741	1.0000
O	064	1.0	0.12400	0.63377	0.24740	1.0000
O	065	1.0	0.74308	0.25114	0.62462	1.0000
O	066	1.0	0.74307	0.74880	0.12462	1.0000
O	067	1.0	0.24307	0.75114	0.62461	1.0000
O	068	1.0	0.24306	0.24880	0.12462	1.0000
O	069	1.0	0.74937	0.74297	0.62651	1.0000
O	070	1.0	0.74938	0.25696	0.12650	1.0000
O	071	1.0	0.24939	0.24295	0.62650	1.0000
O	072	1.0	0.24938	0.75698	0.12650	1.0000
O	073	1.0	0.75926	0.25164	0.87721	1.0000

O	O74	1.0	0.75927	0.74829	0.37721	1.0000
O	O75	1.0	0.25927	0.75165	0.87720	1.0000
O	O76	1.0	0.25927	0.24829	0.37720	1.0000
O	O77	1.0	0.75475	0.75390	0.87694	1.0000
O	O78	1.0	0.75476	0.24605	0.37694	1.0000
O	O79	1.0	0.25474	0.25388	0.87695	1.0000
O	O80	1.0	0.25476	0.74605	0.37694	1.0000
O	O81	1.0	0.01174	0.25007	0.12584	1.0000
O	O82	1.0	0.01174	0.74987	0.62585	1.0000
O	O83	1.0	0.51175	0.75006	0.12584	1.0000
O	O84	1.0	0.51175	0.24987	0.62584	1.0000
O	O85	1.0	0.00448	0.75498	0.12991	1.0000
O	O86	1.0	0.00450	0.24496	0.62991	1.0000
O	O87	1.0	0.50448	0.25498	0.12992	1.0000
O	O88	1.0	0.50448	0.74496	0.62991	1.0000
O	O89	1.0	0.99850	0.24565	0.37774	1.0000
O	O90	1.0	0.99849	0.75429	0.87774	1.0000
O	O91	1.0	0.49850	0.74565	0.37773	1.0000
O	O92	1.0	0.49849	0.25428	0.87774	1.0000
O	O93	1.0	0.99785	0.74520	0.37145	1.0000
O	O94	1.0	0.99785	0.25474	0.87145	1.0000
O	O95	1.0	0.49785	0.24521	0.37145	1.0000
O	O96	1.0	0.49785	0.75473	0.87145	1.0000
O	O97	1.0	0.74278	0.49915	0.62812	1.0000
O	O98	1.0	0.74278	0.50078	0.12811	1.0000
O	O99	1.0	0.24278	0.99913	0.62810	1.0000
O	O100	1.0	0.24277	0.00080	0.12811	1.0000

O	O101	1.0	0.74977	0.00695	0.62128	1.0000
O	O102	1.0	0.74977	0.99299	0.12128	1.0000
O	O103	1.0	0.24977	0.50695	0.62128	1.0000
O	O104	1.0	0.24977	0.49300	0.12128	1.0000
O	O105	1.0	0.75404	0.49374	0.87416	1.0000
O	O106	1.0	0.75405	0.50619	0.37416	1.0000
O	O107	1.0	0.25406	0.99373	0.87415	1.0000
O	O108	1.0	0.25406	0.00620	0.37414	1.0000
O	O109	1.0	0.75462	0.99328	0.88097	1.0000
O	O110	1.0	0.75462	0.00666	0.38098	1.0000
O	O111	1.0	0.25461	0.49327	0.88098	1.0000
O	O112	1.0	0.25461	0.50667	0.38097	1.0000
O	O113	1.0	0.01114	0.00057	0.12293	1.0000
O	O114	1.0	0.01115	0.99936	0.62293	1.0000
O	O115	1.0	0.51115	0.50057	0.12293	1.0000
O	O116	1.0	0.51115	0.49936	0.62293	1.0000
O	O117	1.0	0.00428	0.49377	0.12145	1.0000
O	O118	1.0	0.00427	0.50617	0.62145	1.0000
O	O119	1.0	0.50427	0.99376	0.12144	1.0000
O	O120	1.0	0.50427	0.00618	0.62144	1.0000
O	O121	1.0	0.99387	0.00289	0.37807	1.0000
O	O122	1.0	0.99388	0.99704	0.87806	1.0000
O	O123	1.0	0.49387	0.50289	0.37807	1.0000
O	O124	1.0	0.49386	0.49704	0.87807	1.0000
O	O125	1.0	0.99795	0.50517	0.38155	1.0000
O	O126	1.0	0.99794	0.49477	0.88155	1.0000
O	O127	1.0	0.49795	0.00518	0.38154	1.0000

0	0128	1.0	0.49795	0.99477	0.88155	1.0000

Hematite Supercell CIF

```
data_image0

_chemical_formula_structural       Fe384O576

_chemical_formula_sum              "Fe384 O576"

_cell_length_a       20.3694

_cell_length_b       17.641

_cell_length_c       27.8517

_cell_angle_alpha    89.9993

_cell_angle_beta     90.0007

_cell_angle_gamma    89.9991


_space_group_name_H-M_alt    "P 1"

_space_group_IT_number       1


loop_

  _space_group_symop_operation_xyz

  'x, y, z'


loop_

  _atom_site_type_symbol

  _atom_site_label

  _atom_site_symmetry_multiplicity

  _atom_site_fract_x

  _atom_site_fract_y

  _atom_site_fract_z

  _atom_site_occupancy

  Fe  Fe1       1.0  0.97759  0.61319  0.73808  1.0000
```

Fe	Fe2	1.0	0.97758	0.61308	0.88378	1.0000
Fe	Fe3	1.0	0.22761	0.44656	0.40471	1.0000
Fe	Fe4	1.0	0.47761	0.44654	0.40472	1.0000
Fe	Fe5	1.0	0.10254	0.19656	0.40471	1.0000
Fe	Fe6	1.0	0.35261	0.19657	0.40473	1.0000
Fe	Fe7	1.0	0.22759	0.11326	0.38386	1.0000
Fe	Fe8	1.0	0.47757	0.11321	0.38385	1.0000
Fe	Fe9	1.0	0.10262	0.36319	0.38381	1.0000
Fe	Fe10	1.0	0.35260	0.36324	0.38384	1.0000
Fe	Fe11	1.0	0.22753	0.11325	0.23807	1.0000
Fe	Fe12	1.0	0.47757	0.11325	0.23805	1.0000
Fe	Fe13	1.0	0.10258	0.36318	0.23806	1.0000
Fe	Fe14	1.0	0.35262	0.36327	0.23805	1.0000
Fe	Fe15	1.0	0.35256	0.02994	0.21719	1.0000
Fe	Fe16	1.0	0.10257	0.02985	0.21718	1.0000
Fe	Fe17	1.0	0.22760	0.27991	0.21718	1.0000
Fe	Fe18	1.0	0.47760	0.27991	0.21714	1.0000
Fe	Fe19	1.0	0.35258	0.02990	0.07139	1.0000
Fe	Fe20	1.0	0.10257	0.02980	0.07142	1.0000
Fe	Fe21	1.0	0.22753	0.27983	0.07140	1.0000
Fe	Fe22	1.0	0.47760	0.27985	0.07138	1.0000
Fe	Fe23	1.0	0.22760	0.44650	0.05055	1.0000
Fe	Fe24	1.0	0.47759	0.44653	0.05049	1.0000
Fe	Fe25	1.0	0.10259	0.19645	0.05055	1.0000
Fe	Fe26	1.0	0.35253	0.19655	0.05050	1.0000
Fe	Fe27	1.0	0.72756	0.44662	0.40471	1.0000
Fe	Fe28	1.0	0.97763	0.44658	0.40471	1.0000

Fe	Fe29	1.0	0.60252	0.19656	0.40471	1.0000
Fe	Fe30	1.0	0.85259	0.19663	0.40472	1.0000
Fe	Fe31	1.0	0.72758	0.11329	0.38384	1.0000
Fe	Fe32	1.0	0.97755	0.11322	0.38383	1.0000
Fe	Fe33	1.0	0.60260	0.36322	0.38384	1.0000
Fe	Fe34	1.0	0.85256	0.36330	0.38383	1.0000
Fe	Fe35	1.0	0.72752	0.11328	0.23805	1.0000
Fe	Fe36	1.0	0.97756	0.11320	0.23808	1.0000
Fe	Fe37	1.0	0.60259	0.36324	0.23803	1.0000
Fe	Fe38	1.0	0.85259	0.36326	0.23806	1.0000
Fe	Fe39	1.0	0.85253	0.02990	0.21720	1.0000
Fe	Fe40	1.0	0.60257	0.02989	0.21717	1.0000
Fe	Fe41	1.0	0.72759	0.27992	0.21717	1.0000
Fe	Fe42	1.0	0.97757	0.27986	0.21716	1.0000
Fe	Fe43	1.0	0.85259	0.02983	0.07141	1.0000
Fe	Fe44	1.0	0.60259	0.02986	0.07137	1.0000
Fe	Fe45	1.0	0.72755	0.27983	0.07137	1.0000
Fe	Fe46	1.0	0.97759	0.27976	0.07141	1.0000
Fe	Fe47	1.0	0.72760	0.44647	0.05051	1.0000
Fe	Fe48	1.0	0.97759	0.44644	0.05048	1.0000
Fe	Fe49	1.0	0.60261	0.19650	0.05050	1.0000
Fe	Fe50	1.0	0.85253	0.19648	0.05051	1.0000
Fe	Fe51	1.0	0.22759	0.94659	0.40474	1.0000
Fe	Fe52	1.0	0.47760	0.94655	0.40475	1.0000
Fe	Fe53	1.0	0.10255	0.69656	0.40471	1.0000
Fe	Fe54	1.0	0.35262	0.69659	0.40473	1.0000
Fe	Fe55	1.0	0.22761	0.61324	0.38383	1.0000

Fe	Fe56	1.0	0.47758	0.61321	0.38384	1.0000
Fe	Fe57	1.0	0.10261	0.86319	0.38383	1.0000
Fe	Fe58	1.0	0.35258	0.86327	0.38386	1.0000
Fe	Fe59	1.0	0.22755	0.61325	0.23807	1.0000
Fe	Fe60	1.0	0.47758	0.61326	0.23806	1.0000
Fe	Fe61	1.0	0.10256	0.86318	0.23807	1.0000
Fe	Fe62	1.0	0.35261	0.86328	0.23807	1.0000
Fe	Fe63	1.0	0.35257	0.52994	0.21719	1.0000
Fe	Fe64	1.0	0.10259	0.52984	0.21717	1.0000
Fe	Fe65	1.0	0.22759	0.77992	0.21719	1.0000
Fe	Fe66	1.0	0.47758	0.77991	0.21717	1.0000
Fe	Fe67	1.0	0.35259	0.52990	0.07141	1.0000
Fe	Fe68	1.0	0.10258	0.52981	0.07138	1.0000
Fe	Fe69	1.0	0.22753	0.77984	0.07142	1.0000
Fe	Fe70	1.0	0.47761	0.77985	0.07141	1.0000
Fe	Fe71	1.0	0.22760	0.94650	0.05056	1.0000
Fe	Fe72	1.0	0.47757	0.94653	0.05048	1.0000
Fe	Fe73	1.0	0.10259	0.69646	0.05053	1.0000
Fe	Fe74	1.0	0.35253	0.69656	0.05053	1.0000
Fe	Fe75	1.0	0.72757	0.94662	0.40473	1.0000
Fe	Fe76	1.0	0.97763	0.94658	0.40472	1.0000
Fe	Fe77	1.0	0.60253	0.69657	0.40472	1.0000
Fe	Fe78	1.0	0.85261	0.69663	0.40471	1.0000
Fe	Fe79	1.0	0.72758	0.61329	0.38383	1.0000
Fe	Fe80	1.0	0.97757	0.61322	0.38383	1.0000
Fe	Fe81	1.0	0.60259	0.86323	0.38385	1.0000
Fe	Fe82	1.0	0.85257	0.86330	0.38384	1.0000

Fe	Fe83	1.0	0.72753	0.61328	0.23806	1.0000
Fe	Fe84	1.0	0.97758	0.61319	0.23806	1.0000
Fe	Fe85	1.0	0.60258	0.86324	0.23805	1.0000
Fe	Fe86	1.0	0.85260	0.86326	0.23807	1.0000
Fe	Fe87	1.0	0.85254	0.52990	0.21718	1.0000
Fe	Fe88	1.0	0.60259	0.52988	0.21716	1.0000
Fe	Fe89	1.0	0.72759	0.77993	0.21719	1.0000
Fe	Fe90	1.0	0.97756	0.77984	0.21716	1.0000
Fe	Fe91	1.0	0.85260	0.52981	0.07138	1.0000
Fe	Fe92	1.0	0.60260	0.52987	0.07137	1.0000
Fe	Fe93	1.0	0.72756	0.77984	0.07139	1.0000
Fe	Fe94	1.0	0.97759	0.77977	0.07140	1.0000
Fe	Fe95	1.0	0.72759	0.94648	0.05054	1.0000
Fe	Fe96	1.0	0.97757	0.94644	0.05052	1.0000
Fe	Fe97	1.0	0.60262	0.69650	0.05053	1.0000
Fe	Fe98	1.0	0.85254	0.69646	0.05051	1.0000
Fe	Fe99	1.0	0.22757	0.44650	0.90475	1.0000
Fe	Fe100	1.0	0.47763	0.44650	0.90472	1.0000
Fe	Fe101	1.0	0.10253	0.19643	0.90472	1.0000
Fe	Fe102	1.0	0.35259	0.19653	0.90473	1.0000
Fe	Fe103	1.0	0.22758	0.11320	0.88386	1.0000
Fe	Fe104	1.0	0.47756	0.11316	0.88382	1.0000
Fe	Fe105	1.0	0.10261	0.36310	0.88384	1.0000
Fe	Fe106	1.0	0.35257	0.36321	0.88386	1.0000
Fe	Fe107	1.0	0.22752	0.11325	0.73807	1.0000
Fe	Fe108	1.0	0.47757	0.11321	0.73808	1.0000
Fe	Fe109	1.0	0.10259	0.36317	0.73803	1.0000

Fe	Fe110	1.0	0.35260	0.36328	0.73807	1.0000
Fe	Fe111	1.0	0.35253	0.02994	0.71720	1.0000
Fe	Fe112	1.0	0.10258	0.02989	0.71717	1.0000
Fe	Fe113	1.0	0.22762	0.27994	0.71718	1.0000
Fe	Fe114	1.0	0.47757	0.27990	0.71717	1.0000
Fe	Fe115	1.0	0.35258	0.02993	0.57141	1.0000
Fe	Fe116	1.0	0.10257	0.02992	0.57138	1.0000
Fe	Fe117	1.0	0.22756	0.27994	0.57137	1.0000
Fe	Fe118	1.0	0.47759	0.27988	0.57142	1.0000
Fe	Fe119	1.0	0.22760	0.44659	0.55050	1.0000
Fe	Fe120	1.0	0.47758	0.44658	0.55050	1.0000
Fe	Fe121	1.0	0.10262	0.19658	0.55052	1.0000
Fe	Fe122	1.0	0.35254	0.19660	0.55053	1.0000
Fe	Fe123	1.0	0.72762	0.44645	0.90469	1.0000
Fe	Fe124	1.0	0.97761	0.44638	0.90471	1.0000
Fe	Fe125	1.0	0.60254	0.19649	0.90469	1.0000
Fe	Fe126	1.0	0.85260	0.19642	0.90473	1.0000
Fe	Fe127	1.0	0.72758	0.11316	0.88383	1.0000
Fe	Fe128	1.0	0.97757	0.11310	0.88384	1.0000
Fe	Fe129	1.0	0.60263	0.36315	0.88381	1.0000
Fe	Fe130	1.0	0.85260	0.36310	0.88382	1.0000
Fe	Fe131	1.0	0.72754	0.11324	0.73805	1.0000
Fe	Fe132	1.0	0.97758	0.11318	0.73806	1.0000
Fe	Fe133	1.0	0.60259	0.36320	0.73803	1.0000
Fe	Fe134	1.0	0.85264	0.36322	0.73805	1.0000
Fe	Fe135	1.0	0.85256	0.02992	0.71718	1.0000
Fe	Fe136	1.0	0.60258	0.02987	0.71717	1.0000

Fe	Fe137	1.0	0.72762	0.27994	0.71716	1.0000
Fe	Fe138	1.0	0.97760	0.27986	0.71714	1.0000
Fe	Fe139	1.0	0.85257	0.02997	0.57138	1.0000
Fe	Fe140	1.0	0.60257	0.02992	0.57140	1.0000
Fe	Fe141	1.0	0.72755	0.27994	0.57136	1.0000
Fe	Fe142	1.0	0.97761	0.27990	0.57139	1.0000
Fe	Fe143	1.0	0.72759	0.44661	0.55050	1.0000
Fe	Fe144	1.0	0.97757	0.44659	0.55049	1.0000
Fe	Fe145	1.0	0.60260	0.19657	0.55054	1.0000
Fe	Fe146	1.0	0.85254	0.19664	0.55049	1.0000
Fe	Fe147	1.0	0.22754	0.94650	0.90475	1.0000
Fe	Fe148	1.0	0.47762	0.94648	0.90472	1.0000
Fe	Fe149	1.0	0.10254	0.69644	0.90471	1.0000
Fe	Fe150	1.0	0.35260	0.69651	0.90475	1.0000
Fe	Fe151	1.0	0.22759	0.61320	0.88386	1.0000
Fe	Fe152	1.0	0.47759	0.61316	0.88384	1.0000
Fe	Fe153	1.0	0.10258	0.86311	0.88385	1.0000
Fe	Fe154	1.0	0.35255	0.86319	0.88386	1.0000
Fe	Fe155	1.0	0.22753	0.61326	0.73806	1.0000
Fe	Fe156	1.0	0.47758	0.61321	0.73807	1.0000
Fe	Fe157	1.0	0.10257	0.86321	0.73805	1.0000
Fe	Fe158	1.0	0.35258	0.86325	0.73808	1.0000
Fe	Fe159	1.0	0.35255	0.52996	0.71720	1.0000
Fe	Fe160	1.0	0.10260	0.52986	0.71715	1.0000
Fe	Fe161	1.0	0.22758	0.77994	0.71718	1.0000
Fe	Fe162	1.0	0.47756	0.77989	0.71717	1.0000
Fe	Fe163	1.0	0.35259	0.52996	0.57139	1.0000

Fe	Fe164	1.0	0.10259	0.52992	0.57137	1.0000
Fe	Fe165	1.0	0.22754	0.77994	0.57138	1.0000
Fe	Fe166	1.0	0.47759	0.77988	0.57142	1.0000
Fe	Fe167	1.0	0.22757	0.94659	0.55053	1.0000
Fe	Fe168	1.0	0.47757	0.94657	0.55052	1.0000
Fe	Fe169	1.0	0.10262	0.69657	0.55053	1.0000
Fe	Fe170	1.0	0.35252	0.69660	0.55051	1.0000
Fe	Fe171	1.0	0.72760	0.94647	0.90471	1.0000
Fe	Fe172	1.0	0.97760	0.94642	0.90475	1.0000
Fe	Fe173	1.0	0.60257	0.69649	0.90470	1.0000
Fe	Fe174	1.0	0.85261	0.69645	0.90473	1.0000
Fe	Fe175	1.0	0.72761	0.61313	0.88383	1.0000
Fe	Fe176	1.0	0.60261	0.86312	0.88383	1.0000
Fe	Fe177	1.0	0.85257	0.86312	0.88385	1.0000
Fe	Fe178	1.0	0.72754	0.61324	0.73804	1.0000
Fe	Fe179	1.0	0.60257	0.86319	0.73805	1.0000
Fe	Fe180	1.0	0.85262	0.86324	0.73806	1.0000
Fe	Fe181	1.0	0.85256	0.52992	0.71715	1.0000
Fe	Fe182	1.0	0.60260	0.52987	0.71716	1.0000
Fe	Fe183	1.0	0.72761	0.77993	0.71717	1.0000
Fe	Fe184	1.0	0.97758	0.77988	0.71716	1.0000
Fe	Fe185	1.0	0.85258	0.52997	0.57138	1.0000
Fe	Fe186	1.0	0.60259	0.52992	0.57138	1.0000
Fe	Fe187	1.0	0.72755	0.77994	0.57138	1.0000
Fe	Fe188	1.0	0.97760	0.77990	0.57139	1.0000
Fe	Fe189	1.0	0.72759	0.94661	0.55052	1.0000
Fe	Fe190	1.0	0.97755	0.94660	0.55049	1.0000

Fe	Fe191	1.0	0.60261	0.69657	0.55053	1.0000
Fe	Fe192	1.0	0.85254	0.69663	0.55050	1.0000
Fe	Fe193	1.0	0.97759	0.61325	0.63386	1.0000
Fe	Fe194	1.0	0.35261	0.02992	0.46719	1.0000
Fe	Fe195	1.0	0.10257	0.02995	0.46716	1.0000
Fe	Fe196	1.0	0.22757	0.27987	0.46716	1.0000
Fe	Fe197	1.0	0.47759	0.27993	0.46720	1.0000
Fe	Fe198	1.0	0.35255	0.02991	0.32142	1.0000
Fe	Fe199	1.0	0.10261	0.02989	0.32140	1.0000
Fe	Fe200	1.0	0.22762	0.27987	0.32139	1.0000
Fe	Fe201	1.0	0.47757	0.27993	0.32137	1.0000
Fe	Fe202	1.0	0.22762	0.44654	0.30049	1.0000
Fe	Fe203	1.0	0.47762	0.44661	0.30049	1.0000
Fe	Fe204	1.0	0.10254	0.19655	0.30050	1.0000
Fe	Fe205	1.0	0.35258	0.19657	0.30049	1.0000
Fe	Fe206	1.0	0.22762	0.44654	0.15474	1.0000
Fe	Fe207	1.0	0.47757	0.44656	0.15471	1.0000
Fe	Fe208	1.0	0.10258	0.19651	0.15475	1.0000
Fe	Fe209	1.0	0.35253	0.19654	0.15471	1.0000
Fe	Fe210	1.0	0.22755	0.11318	0.13386	1.0000
Fe	Fe211	1.0	0.47758	0.11323	0.13383	1.0000
Fe	Fe212	1.0	0.10259	0.36318	0.13386	1.0000
Fe	Fe213	1.0	0.35262	0.36319	0.13383	1.0000
Fe	Fe214	1.0	0.22759	0.11322	0.48807	1.0000
Fe	Fe215	1.0	0.47758	0.11328	0.48809	1.0000
Fe	Fe216	1.0	0.10261	0.36326	0.48802	1.0000
Fe	Fe217	1.0	0.35257	0.36324	0.48807	1.0000

Fe	Fe218	1.0	0.85260	0.02996	0.46716	1.0000
Fe	Fe219	1.0	0.60255	0.02993	0.46718	1.0000
Fe	Fe220	1.0	0.72754	0.27990	0.46714	1.0000
Fe	Fe221	1.0	0.97758	0.27999	0.46718	1.0000
Fe	Fe222	1.0	0.85255	0.02992	0.32140	1.0000
Fe	Fe223	1.0	0.60260	0.02992	0.32139	1.0000
Fe	Fe224	1.0	0.72758	0.27992	0.32138	1.0000
Fe	Fe225	1.0	0.97755	0.27991	0.32138	1.0000
Fe	Fe226	1.0	0.72758	0.44659	0.30049	1.0000
Fe	Fe227	1.0	0.97760	0.44658	0.30050	1.0000
Fe	Fe228	1.0	0.60253	0.19659	0.30049	1.0000
Fe	Fe229	1.0	0.85254	0.19657	0.30049	1.0000
Fe	Fe230	1.0	0.72762	0.44654	0.15472	1.0000
Fe	Fe231	1.0	0.97755	0.44649	0.15471	1.0000
Fe	Fe232	1.0	0.60259	0.19656	0.15471	1.0000
Fe	Fe233	1.0	0.85253	0.19649	0.15473	1.0000
Fe	Fe234	1.0	0.72754	0.11318	0.13384	1.0000
Fe	Fe235	1.0	0.97758	0.11315	0.13388	1.0000
Fe	Fe236	1.0	0.60260	0.36324	0.13382	1.0000
Fe	Fe237	1.0	0.85260	0.36313	0.13383	1.0000
Fe	Fe238	1.0	0.72756	0.11324	0.48805	1.0000
Fe	Fe239	1.0	0.97759	0.11332	0.48807	1.0000
Fe	Fe240	1.0	0.60259	0.36326	0.48806	1.0000
Fe	Fe241	1.0	0.85254	0.36329	0.48805	1.0000
Fe	Fe242	1.0	0.35262	0.52992	0.46717	1.0000
Fe	Fe243	1.0	0.10260	0.52993	0.46714	1.0000
Fe	Fe244	1.0	0.22754	0.77987	0.46716	1.0000

Fe	Fe245	1.0	0.47759	0.77994	0.46721	1.0000
Fe	Fe246	1.0	0.35258	0.52990	0.32140	1.0000
Fe	Fe247	1.0	0.10262	0.52987	0.32138	1.0000
Fe	Fe248	1.0	0.22762	0.77990	0.32140	1.0000
Fe	Fe249	1.0	0.47757	0.77995	0.32139	1.0000
Fe	Fe250	1.0	0.22759	0.94656	0.30051	1.0000
Fe	Fe251	1.0	0.47761	0.94663	0.30052	1.0000
Fe	Fe252	1.0	0.10255	0.69655	0.30050	1.0000
Fe	Fe253	1.0	0.35258	0.69657	0.30049	1.0000
Fe	Fe254	1.0	0.22760	0.94653	0.15475	1.0000
Fe	Fe255	1.0	0.47755	0.94654	0.15471	1.0000
Fe	Fe256	1.0	0.10258	0.69651	0.15474	1.0000
Fe	Fe257	1.0	0.35253	0.69655	0.15474	1.0000
Fe	Fe258	1.0	0.22755	0.61318	0.13387	1.0000
Fe	Fe259	1.0	0.47760	0.61324	0.13385	1.0000
Fe	Fe260	1.0	0.10258	0.86317	0.13388	1.0000
Fe	Fe261	1.0	0.35260	0.86320	0.13385	1.0000
Fe	Fe262	1.0	0.22760	0.61321	0.48804	1.0000
Fe	Fe263	1.0	0.47759	0.61328	0.48808	1.0000
Fe	Fe264	1.0	0.10260	0.86328	0.48804	1.0000
Fe	Fe265	1.0	0.35254	0.86323	0.48808	1.0000
Fe	Fe266	1.0	0.85260	0.52997	0.46715	1.0000
Fe	Fe267	1.0	0.60256	0.52992	0.46716	1.0000
Fe	Fe268	1.0	0.72754	0.77990	0.46716	1.0000
Fe	Fe269	1.0	0.97758	0.77998	0.46718	1.0000
Fe	Fe270	1.0	0.85255	0.52992	0.32139	1.0000
Fe	Fe271	1.0	0.60260	0.52993	0.32138	1.0000

Fe	Fe272	1.0	0.72759	0.77993	0.32140	1.0000
Fe	Fe273	1.0	0.97756	0.77991	0.32138	1.0000
Fe	Fe274	1.0	0.72758	0.94659	0.30051	1.0000
Fe	Fe275	1.0	0.97760	0.94657	0.30051	1.0000
Fe	Fe276	1.0	0.60253	0.69660	0.30051	1.0000
Fe	Fe277	1.0	0.85256	0.69657	0.30049	1.0000
Fe	Fe278	1.0	0.72762	0.94654	0.15474	1.0000
Fe	Fe279	1.0	0.97754	0.94648	0.15474	1.0000
Fe	Fe280	1.0	0.60260	0.69658	0.15474	1.0000
Fe	Fe281	1.0	0.85252	0.69649	0.15472	1.0000
Fe	Fe282	1.0	0.72755	0.61317	0.13385	1.0000
Fe	Fe283	1.0	0.97758	0.61316	0.13384	1.0000
Fe	Fe284	1.0	0.60259	0.86325	0.13385	1.0000
Fe	Fe285	1.0	0.85259	0.86313	0.13385	1.0000
Fe	Fe286	1.0	0.72757	0.61325	0.48805	1.0000
Fe	Fe287	1.0	0.97761	0.61331	0.48807	1.0000
Fe	Fe288	1.0	0.60259	0.86327	0.48807	1.0000
Fe	Fe289	1.0	0.85254	0.86326	0.48806	1.0000
Fe	Fe290	1.0	0.35259	0.02983	0.96717	1.0000
Fe	Fe291	1.0	0.10256	0.02981	0.96720	1.0000
Fe	Fe292	1.0	0.22755	0.27976	0.96719	1.0000
Fe	Fe293	1.0	0.47759	0.27989	0.96717	1.0000
Fe	Fe294	1.0	0.35256	0.02986	0.82142	1.0000
Fe	Fe295	1.0	0.10260	0.02983	0.82139	1.0000
Fe	Fe296	1.0	0.22760	0.27985	0.82140	1.0000
Fe	Fe297	1.0	0.47755	0.27989	0.82138	1.0000
Fe	Fe298	1.0	0.22758	0.44653	0.80051	1.0000

Fe	Fe299	1.0	0.47762	0.44657	0.80051	1.0000
Fe	Fe300	1.0	0.10255	0.19652	0.80050	1.0000
Fe	Fe301	1.0	0.35256	0.19655	0.80049	1.0000
Fe	Fe302	1.0	0.22763	0.44661	0.65471	1.0000
Fe	Fe303	1.0	0.47756	0.44655	0.65473	1.0000
Fe	Fe304	1.0	0.10260	0.19658	0.65472	1.0000
Fe	Fe305	1.0	0.35253	0.19656	0.65474	1.0000
Fe	Fe306	1.0	0.22754	0.11324	0.63385	1.0000
Fe	Fe307	1.0	0.47758	0.11324	0.63388	1.0000
Fe	Fe308	1.0	0.10262	0.36328	0.63382	1.0000
Fe	Fe309	1.0	0.35259	0.36324	0.63385	1.0000
Fe	Fe310	1.0	0.22757	0.11311	0.98808	1.0000
Fe	Fe311	1.0	0.47758	0.11322	0.98806	1.0000
Fe	Fe312	1.0	0.10262	0.36316	0.98806	1.0000
Fe	Fe313	1.0	0.35253	0.36319	0.98808	1.0000
Fe	Fe314	1.0	0.85260	0.02973	0.96719	1.0000
Fe	Fe315	1.0	0.60257	0.02985	0.96715	1.0000
Fe	Fe316	1.0	0.72755	0.27975	0.96716	1.0000
Fe	Fe317	1.0	0.97760	0.27978	0.96719	1.0000
Fe	Fe318	1.0	0.85257	0.02980	0.82141	1.0000
Fe	Fe319	1.0	0.60261	0.02982	0.82138	1.0000
Fe	Fe320	1.0	0.72761	0.27980	0.82137	1.0000
Fe	Fe321	1.0	0.97757	0.27982	0.82136	1.0000
Fe	Fe322	1.0	0.72763	0.44651	0.80047	1.0000
Fe	Fe323	1.0	0.97764	0.44652	0.80048	1.0000
Fe	Fe324	1.0	0.60254	0.19654	0.80049	1.0000
Fe	Fe325	1.0	0.85257	0.19649	0.80048	1.0000

Fe	Fe326	1.0	0.72763	0.44659	0.65470	1.0000
Fe	Fe327	1.0	0.97758	0.44655	0.65471	1.0000
Fe	Fe328	1.0	0.60258	0.19659	0.65474	1.0000
Fe	Fe329	1.0	0.85255	0.19655	0.65470	1.0000
Fe	Fe330	1.0	0.72754	0.11326	0.63384	1.0000
Fe	Fe331	1.0	0.97759	0.11325	0.63384	1.0000
Fe	Fe332	1.0	0.60261	0.36328	0.63383	1.0000
Fe	Fe333	1.0	0.85261	0.36323	0.63382	1.0000
Fe	Fe334	1.0	0.72760	0.11307	0.98805	1.0000
Fe	Fe335	1.0	0.97756	0.11314	0.98810	1.0000
Fe	Fe336	1.0	0.60262	0.36320	0.98803	1.0000
Fe	Fe337	1.0	0.85255	0.36306	0.98805	1.0000
Fe	Fe338	1.0	0.35262	0.52986	0.96720	1.0000
Fe	Fe339	1.0	0.10255	0.52982	0.96716	1.0000
Fe	Fe340	1.0	0.22753	0.77977	0.96719	1.0000
Fe	Fe341	1.0	0.47760	0.77990	0.96719	1.0000
Fe	Fe342	1.0	0.35256	0.52987	0.82142	1.0000
Fe	Fe343	1.0	0.10262	0.52983	0.82138	1.0000
Fe	Fe344	1.0	0.22758	0.77985	0.82140	1.0000
Fe	Fe345	1.0	0.47755	0.77987	0.82139	1.0000
Fe	Fe346	1.0	0.22758	0.94654	0.80052	1.0000
Fe	Fe347	1.0	0.47760	0.94656	0.80050	1.0000
Fe	Fe348	1.0	0.10254	0.69654	0.80050	1.0000
Fe	Fe349	1.0	0.35256	0.69655	0.80051	1.0000
Fe	Fe350	1.0	0.22759	0.94656	0.65473	1.0000
Fe	Fe351	1.0	0.47754	0.94654	0.65474	1.0000
Fe	Fe352	1.0	0.10258	0.69658	0.65473	1.0000

Fe	Fe353	1.0	0.35252	0.69656	0.65473	1.0000
Fe	Fe354	1.0	0.22754	0.61326	0.63385	1.0000
Fe	Fe355	1.0	0.47759	0.61325	0.63386	1.0000
Fe	Fe356	1.0	0.10257	0.86328	0.63384	1.0000
Fe	Fe357	1.0	0.35258	0.86322	0.63385	1.0000
Fe	Fe358	1.0	0.22758	0.61311	0.98809	1.0000
Fe	Fe359	1.0	0.47760	0.61324	0.98808	1.0000
Fe	Fe360	1.0	0.10259	0.86317	0.98807	1.0000
Fe	Fe361	1.0	0.35252	0.86315	0.98808	1.0000
Fe	Fe362	1.0	0.85262	0.52973	0.96715	1.0000
Fe	Fe363	1.0	0.60261	0.52986	0.96715	1.0000
Fe	Fe364	1.0	0.72755	0.77975	0.96717	1.0000
Fe	Fe365	1.0	0.97759	0.77978	0.96718	1.0000
Fe	Fe366	1.0	0.85261	0.52981	0.82137	1.0000
Fe	Fe367	1.0	0.60264	0.52983	0.82137	1.0000
Fe	Fe368	1.0	0.72761	0.77980	0.82138	1.0000
Fe	Fe369	1.0	0.97755	0.77985	0.82138	1.0000
Fe	Fe370	1.0	0.72760	0.94650	0.80049	1.0000
Fe	Fe371	1.0	0.97761	0.94652	0.80053	1.0000
Fe	Fe372	1.0	0.60254	0.69654	0.80049	1.0000
Fe	Fe373	1.0	0.85257	0.69650	0.80047	1.0000
Fe	Fe374	1.0	0.72762	0.94658	0.65472	1.0000
Fe	Fe375	1.0	0.97755	0.94655	0.65471	1.0000
Fe	Fe376	1.0	0.60259	0.69659	0.65473	1.0000
Fe	Fe377	1.0	0.85255	0.69656	0.65471	1.0000
Fe	Fe378	1.0	0.72755	0.61326	0.63384	1.0000
Fe	Fe379	1.0	0.60260	0.86328	0.63385	1.0000

Fe	Fe380	1.0	0.85259	0.86323	0.63383	1.0000
Fe	Fe381	1.0	0.72762	0.61308	0.98805	1.0000
Fe	Fe382	1.0	0.97757	0.61314	0.98802	1.0000
Fe	Fe383	1.0	0.60263	0.86319	0.98805	1.0000
Fe	Fe384	1.0	0.85255	0.86308	0.98808	1.0000
O	O1	1.0	0.18939	0.03684	0.43599	1.0000
O	O2	1.0	0.43944	0.03682	0.43599	1.0000
O	O3	1.0	0.30399	0.11326	0.43597	1.0000
O	O4	1.0	0.05392	0.11329	0.43596	1.0000
O	O5	1.0	0.18930	0.18962	0.43596	1.0000
O	O6	1.0	0.43939	0.18967	0.43598	1.0000
O	O7	1.0	0.06439	0.28685	0.43596	1.0000
O	O8	1.0	0.31435	0.28680	0.43594	1.0000
O	O9	1.0	0.17906	0.36322	0.43591	1.0000
O	O10	1.0	0.42896	0.36325	0.43598	1.0000
O	O11	1.0	0.06448	0.43966	0.43589	1.0000
O	O12	1.0	0.31443	0.43967	0.43593	1.0000
O	O13	1.0	0.26576	0.02292	0.35265	1.0000
O	O14	1.0	0.01581	0.02295	0.35263	1.0000
O	O15	1.0	0.14079	0.12018	0.35262	1.0000
O	O16	1.0	0.39079	0.12017	0.35262	1.0000
O	O17	1.0	0.27626	0.19655	0.35263	1.0000
O	O18	1.0	0.02612	0.19655	0.35259	1.0000
O	O19	1.0	0.14080	0.27288	0.35260	1.0000
O	O20	1.0	0.39082	0.27296	0.35263	1.0000
O	O21	1.0	0.01581	0.37018	0.35259	1.0000
O	O22	1.0	0.26582	0.37015	0.35261	1.0000

O	O23	1.0	0.15119	0.44654	0.35261	1.0000
O	O24	1.0	0.40124	0.44657	0.35261	1.0000
O	O25	1.0	0.31441	0.45353	0.26929	1.0000
O	O26	1.0	0.06443	0.45349	0.26929	1.0000
O	O27	1.0	0.42896	0.02996	0.26931	1.0000
O	O28	1.0	0.17903	0.02989	0.26929	1.0000
O	O29	1.0	0.31431	0.10636	0.26930	1.0000
O	O30	1.0	0.06438	0.10629	0.26929	1.0000
O	O31	1.0	0.18933	0.20350	0.26931	1.0000
O	O32	1.0	0.43933	0.20352	0.26926	1.0000
O	O33	1.0	0.30399	0.27994	0.26928	1.0000
O	O34	1.0	0.05389	0.27988	0.26928	1.0000
O	O35	1.0	0.18938	0.35634	0.26927	1.0000
O	O36	1.0	0.43945	0.35636	0.26924	1.0000
O	O37	1.0	0.26578	0.03679	0.18597	1.0000
O	O38	1.0	0.01576	0.03670	0.18598	1.0000
O	O39	1.0	0.15116	0.11321	0.18597	1.0000
O	O40	1.0	0.40120	0.11328	0.18596	1.0000
O	O41	1.0	0.26573	0.18963	0.18595	1.0000
O	O42	1.0	0.01583	0.18957	0.18602	1.0000
O	O43	1.0	0.14079	0.28673	0.18598	1.0000
O	O44	1.0	0.39080	0.28679	0.18591	1.0000
O	O45	1.0	0.02620	0.36322	0.18595	1.0000
O	O46	1.0	0.27622	0.36326	0.18597	1.0000
O	O47	1.0	0.14083	0.43957	0.18597	1.0000
O	O48	1.0	0.39077	0.43966	0.18598	1.0000
O	O49	1.0	0.30405	0.44656	0.10266	1.0000

O	050	1.0	0.05402	0.44647	0.10259	1.0000
O	051	1.0	0.18936	0.02290	0.10266	1.0000
O	052	1.0	0.43944	0.02298	0.10257	1.0000
O	053	1.0	0.31431	0.12015	0.10259	1.0000
O	054	1.0	0.06438	0.12005	0.10266	1.0000
O	055	1.0	0.17896	0.19647	0.10263	1.0000
O	056	1.0	0.42893	0.19653	0.10262	1.0000
O	057	1.0	0.06438	0.27290	0.10266	1.0000
O	058	1.0	0.31431	0.27293	0.10263	1.0000
O	059	1.0	0.18940	0.37007	0.10267	1.0000
O	060	1.0	0.43946	0.37014	0.10261	1.0000
O	061	1.0	0.39070	0.45348	0.01934	1.0000
O	062	1.0	0.14083	0.45342	0.01929	1.0000
O	063	1.0	0.27618	0.02978	0.01930	1.0000
O	064	1.0	0.02618	0.02975	0.01929	1.0000
O	065	1.0	0.39075	0.10633	0.01926	1.0000
O	066	1.0	0.14077	0.10622	0.01931	1.0000
O	067	1.0	0.26573	0.20342	0.01929	1.0000
O	068	1.0	0.01579	0.20336	0.01937	1.0000
O	069	1.0	0.15115	0.27976	0.01929	1.0000
O	070	1.0	0.40121	0.27985	0.01930	1.0000
O	071	1.0	0.26575	0.35625	0.01933	1.0000
O	072	1.0	0.01581	0.35623	0.01927	1.0000
O	073	1.0	0.68934	0.03687	0.43596	1.0000
O	074	1.0	0.93944	0.03685	0.43596	1.0000
O	075	1.0	0.80397	0.11331	0.43595	1.0000
O	076	1.0	0.55391	0.11326	0.43596	1.0000

O	O77	1.0	0.68929	0.18964	0.43594	1.0000
O	O78	1.0	0.93938	0.18973	0.43595	1.0000
O	O79	1.0	0.56439	0.28682	0.43597	1.0000
O	O80	1.0	0.81430	0.28686	0.43591	1.0000
O	O81	1.0	0.67902	0.36329	0.43594	1.0000
O	O82	1.0	0.92894	0.36332	0.43596	1.0000
O	O83	1.0	0.56441	0.43962	0.43594	1.0000
O	O84	1.0	0.81440	0.43974	0.43592	1.0000
O	O85	1.0	0.76573	0.02293	0.35263	1.0000
O	O86	1.0	0.51582	0.02296	0.35264	1.0000
O	O87	1.0	0.64077	0.12022	0.35261	1.0000
O	O88	1.0	0.89077	0.12019	0.35261	1.0000
O	O89	1.0	0.77625	0.19658	0.35261	1.0000
O	O90	1.0	0.52612	0.19655	0.35259	1.0000
O	O91	1.0	0.64077	0.27293	0.35259	1.0000
O	O92	1.0	0.89080	0.27300	0.35263	1.0000
O	O93	1.0	0.51581	0.37019	0.35260	1.0000
O	O94	1.0	0.76575	0.37022	0.35260	1.0000
O	O95	1.0	0.65112	0.44660	0.35264	1.0000
O	O96	1.0	0.90121	0.44661	0.35261	1.0000
O	O97	1.0	0.81438	0.45354	0.26929	1.0000
O	O98	1.0	0.56444	0.45353	0.26928	1.0000
O	O99	1.0	0.92895	0.02990	0.26932	1.0000
O	O100	1.0	0.67902	0.02993	0.26928	1.0000
O	O101	1.0	0.81428	0.10636	0.26929	1.0000
O	O102	1.0	0.56437	0.10631	0.26927	1.0000
O	O103	1.0	0.68931	0.20355	0.26929	1.0000

O	O104	1.0	0.93932	0.20348	0.26926	1.0000
O	O105	1.0	0.80396	0.27998	0.26931	1.0000
O	O106	1.0	0.55391	0.27992	0.26925	1.0000
O	O107	1.0	0.68937	0.35640	0.26925	1.0000
O	O108	1.0	0.93940	0.35633	0.26927	1.0000
O	O109	1.0	0.76575	0.03679	0.18596	1.0000
O	O110	1.0	0.51576	0.03678	0.18593	1.0000
O	O111	1.0	0.65116	0.11326	0.18595	1.0000
O	O112	1.0	0.90118	0.11324	0.18599	1.0000
O	O113	1.0	0.76572	0.18964	0.18594	1.0000
O	O114	1.0	0.51585	0.18963	0.18598	1.0000
O	O115	1.0	0.64079	0.28679	0.18595	1.0000
O	O116	1.0	0.89075	0.28672	0.18595	1.0000
O	O117	1.0	0.52622	0.36325	0.18592	1.0000
O	O118	1.0	0.77621	0.36325	0.18596	1.0000
O	O119	1.0	0.64084	0.43964	0.18594	1.0000
O	O120	1.0	0.89073	0.43960	0.18597	1.0000
O	O121	1.0	0.80405	0.44653	0.10265	1.0000
O	O122	1.0	0.55403	0.44655	0.10258	1.0000
O	O123	1.0	0.68936	0.02293	0.10262	1.0000
O	O124	1.0	0.93943	0.02288	0.10263	1.0000
O	O125	1.0	0.81430	0.12009	0.10260	1.0000
O	O126	1.0	0.56438	0.12012	0.10263	1.0000
O	O127	1.0	0.67895	0.19650	0.10260	1.0000
O	O128	1.0	0.92892	0.19646	0.10265	1.0000
O	O129	1.0	0.56439	0.27299	0.10261	1.0000
O	O130	1.0	0.81431	0.27289	0.10262	1.0000

O	O131	1.0	0.68941	0.37009	0.10262	1.0000
O	O132	1.0	0.93945	0.37006	0.10263	1.0000
O	O133	1.0	0.89071	0.45334	0.01932	1.0000
O	O134	1.0	0.64084	0.45345	0.01925	1.0000
O	O135	1.0	0.77619	0.02972	0.01929	1.0000
O	O136	1.0	0.52619	0.02981	0.01924	1.0000
O	O137	1.0	0.89074	0.10624	0.01929	1.0000
O	O138	1.0	0.64077	0.10626	0.01925	1.0000
O	O139	1.0	0.76574	0.20338	0.01927	1.0000
O	O140	1.0	0.51582	0.20345	0.01933	1.0000
O	O141	1.0	0.65115	0.27980	0.01925	1.0000
O	O142	1.0	0.90121	0.27978	0.01932	1.0000
O	O143	1.0	0.76576	0.35621	0.01928	1.0000
O	O144	1.0	0.51581	0.35633	0.01924	1.0000
O	O145	1.0	0.18940	0.53682	0.43594	1.0000
O	O146	1.0	0.43945	0.53681	0.43597	1.0000
O	O147	1.0	0.30401	0.61327	0.43595	1.0000
O	O148	1.0	0.05392	0.61328	0.43595	1.0000
O	O149	1.0	0.18931	0.68961	0.43594	1.0000
O	O150	1.0	0.43940	0.68969	0.43596	1.0000
O	O151	1.0	0.06439	0.78684	0.43596	1.0000
O	O152	1.0	0.31431	0.78681	0.43594	1.0000
O	O153	1.0	0.17903	0.86324	0.43594	1.0000
O	O154	1.0	0.42894	0.86324	0.43599	1.0000
O	O155	1.0	0.06445	0.93965	0.43592	1.0000
O	O156	1.0	0.31440	0.93966	0.43597	1.0000
O	O157	1.0	0.26578	0.52290	0.35262	1.0000

O	O158	1.0	0.01582	0.52294	0.35262	1.0000
O	O159	1.0	0.14079	0.62016	0.35261	1.0000
O	O160	1.0	0.39080	0.62018	0.35261	1.0000
O	O161	1.0	0.27628	0.69656	0.35262	1.0000
O	O162	1.0	0.02614	0.69654	0.35259	1.0000
O	O163	1.0	0.14082	0.77289	0.35262	1.0000
O	O164	1.0	0.39081	0.77299	0.35264	1.0000
O	O165	1.0	0.01581	0.87018	0.35260	1.0000
O	O166	1.0	0.26579	0.87021	0.35263	1.0000
O	O167	1.0	0.15116	0.94658	0.35265	1.0000
O	O168	1.0	0.40121	0.94663	0.35267	1.0000
O	O169	1.0	0.31440	0.95355	0.26931	1.0000
O	O170	1.0	0.06443	0.95349	0.26931	1.0000
O	O171	1.0	0.42897	0.52996	0.26931	1.0000
O	O172	1.0	0.17904	0.52987	0.26927	1.0000
O	O173	1.0	0.31432	0.60636	0.26930	1.0000
O	O174	1.0	0.06439	0.60627	0.26927	1.0000
O	O175	1.0	0.18934	0.70350	0.26932	1.0000
O	O176	1.0	0.43934	0.70353	0.26927	1.0000
O	O177	1.0	0.30398	0.77997	0.26932	1.0000
O	O178	1.0	0.05390	0.77986	0.26929	1.0000
O	O179	1.0	0.18936	0.85635	0.26928	1.0000
O	O180	1.0	0.43941	0.85638	0.26928	1.0000
O	O181	1.0	0.26579	0.53679	0.18596	1.0000
O	O182	1.0	0.01578	0.53671	0.18595	1.0000
O	O183	1.0	0.15117	0.61322	0.18597	1.0000
O	O184	1.0	0.40121	0.61329	0.18597	1.0000

O	O185	1.0	0.26574	0.68963	0.18598	1.0000
O	O186	1.0	0.01584	0.68956	0.18600	1.0000
O	O187	1.0	0.14078	0.78672	0.18599	1.0000
O	O188	1.0	0.39077	0.78679	0.18595	1.0000
O	O189	1.0	0.02618	0.86320	0.18596	1.0000
O	O190	1.0	0.27621	0.86326	0.18598	1.0000
O	O191	1.0	0.14081	0.93958	0.18598	1.0000
O	O192	1.0	0.39075	0.93966	0.18599	1.0000
O	O193	1.0	0.30405	0.94657	0.10266	1.0000
O	O194	1.0	0.05400	0.94646	0.10263	1.0000
O	O195	1.0	0.18935	0.52291	0.10265	1.0000
O	O196	1.0	0.43946	0.52298	0.10259	1.0000
O	O197	1.0	0.31432	0.62014	0.10263	1.0000
O	O198	1.0	0.06439	0.62006	0.10264	1.0000
O	O199	1.0	0.17895	0.69649	0.10264	1.0000
O	O200	1.0	0.42895	0.69655	0.10264	1.0000
O	O201	1.0	0.06438	0.77290	0.10265	1.0000
O	O202	1.0	0.31431	0.77294	0.10265	1.0000
O	O203	1.0	0.18939	0.87007	0.10269	1.0000
O	O204	1.0	0.43945	0.87015	0.10263	1.0000
O	O205	1.0	0.39068	0.95345	0.01932	1.0000
O	O206	1.0	0.14081	0.95341	0.01933	1.0000
O	O207	1.0	0.27618	0.52980	0.01932	1.0000
O	O208	1.0	0.02618	0.52975	0.01924	1.0000
O	O209	1.0	0.39076	0.60635	0.01928	1.0000
O	O210	1.0	0.14075	0.60624	0.01928	1.0000
O	O211	1.0	0.26570	0.70343	0.01933	1.0000

O	O212	1.0	0.01580	0.70335	0.01933	1.0000
O	O213	1.0	0.15115	0.77977	0.01928	1.0000
O	O214	1.0	0.40121	0.77985	0.01932	1.0000
O	O215	1.0	0.26575	0.85625	0.01933	1.0000
O	O216	1.0	0.01578	0.85623	0.01928	1.0000
O	O217	1.0	0.68935	0.53688	0.43594	1.0000
O	O218	1.0	0.93945	0.53685	0.43595	1.0000
O	O219	1.0	0.80398	0.61332	0.43594	1.0000
O	O220	1.0	0.55392	0.61327	0.43596	1.0000
O	O221	1.0	0.68929	0.68965	0.43595	1.0000
O	O222	1.0	0.93939	0.68973	0.43594	1.0000
O	O223	1.0	0.56440	0.78683	0.43599	1.0000
O	O224	1.0	0.81430	0.78686	0.43592	1.0000
O	O225	1.0	0.67902	0.86329	0.43595	1.0000
O	O226	1.0	0.92894	0.86330	0.43597	1.0000
O	O227	1.0	0.56440	0.93962	0.43597	1.0000
O	O228	1.0	0.81439	0.93971	0.43594	1.0000
O	O229	1.0	0.76574	0.52294	0.35262	1.0000
O	O230	1.0	0.51582	0.52295	0.35263	1.0000
O	O231	1.0	0.64078	0.62023	0.35261	1.0000
O	O232	1.0	0.89078	0.62019	0.35260	1.0000
O	O233	1.0	0.77626	0.69658	0.35261	1.0000
O	O234	1.0	0.52613	0.69658	0.35260	1.0000
O	O235	1.0	0.64079	0.77293	0.35263	1.0000
O	O236	1.0	0.89080	0.77300	0.35263	1.0000
O	O237	1.0	0.51579	0.87021	0.35263	1.0000
O	O238	1.0	0.76575	0.87022	0.35263	1.0000

O	O239	1.0	0.65111	0.94660	0.35265	1.0000
O	O240	1.0	0.90121	0.94662	0.35263	1.0000
O	O241	1.0	0.81439	0.95354	0.26931	1.0000
O	O242	1.0	0.56443	0.95353	0.26929	1.0000
O	O243	1.0	0.92895	0.52991	0.26930	1.0000
O	O244	1.0	0.67902	0.52993	0.26928	1.0000
O	O245	1.0	0.81430	0.60636	0.26928	1.0000
O	O246	1.0	0.56438	0.60632	0.26928	1.0000
O	O247	1.0	0.68932	0.70355	0.26932	1.0000
O	O248	1.0	0.93932	0.70346	0.26926	1.0000
O	O249	1.0	0.80396	0.77997	0.26933	1.0000
O	O250	1.0	0.55391	0.77995	0.26929	1.0000
O	O251	1.0	0.68937	0.85640	0.26926	1.0000
O	O252	1.0	0.93941	0.85631	0.26928	1.0000
O	O253	1.0	0.76576	0.53678	0.18596	1.0000
O	O254	1.0	0.51578	0.53678	0.18594	1.0000
O	O255	1.0	0.65117	0.61326	0.18597	1.0000
O	O256	1.0	0.90120	0.61324	0.18597	1.0000
O	O257	1.0	0.76573	0.68963	0.18596	1.0000
O	O258	1.0	0.51587	0.68965	0.18601	1.0000
O	O259	1.0	0.64080	0.78681	0.18598	1.0000
O	O260	1.0	0.89073	0.78671	0.18596	1.0000
O	O261	1.0	0.52620	0.86325	0.18594	1.0000
O	O262	1.0	0.77621	0.86325	0.18597	1.0000
O	O263	1.0	0.64082	0.93964	0.18596	1.0000
O	O264	1.0	0.89073	0.93959	0.18600	1.0000
O	O265	1.0	0.80405	0.94653	0.10267	1.0000

O	O266	1.0	0.55402	0.94654	0.10259	1.0000
O	O267	1.0	0.68938	0.52293	0.10261	1.0000
O	O268	1.0	0.93945	0.52290	0.10258	1.0000
O	O269	1.0	0.81431	0.62008	0.10260	1.0000
O	O270	1.0	0.56440	0.62013	0.10264	1.0000
O	O271	1.0	0.67896	0.69650	0.10263	1.0000
O	O272	1.0	0.92892	0.69647	0.10264	1.0000
O	O273	1.0	0.56439	0.77298	0.10266	1.0000
O	O274	1.0	0.81432	0.77288	0.10263	1.0000
O	O275	1.0	0.68941	0.87009	0.10266	1.0000
O	O276	1.0	0.93943	0.87004	0.10264	1.0000
O	O277	1.0	0.89070	0.95335	0.01935	1.0000
O	O278	1.0	0.64083	0.95345	0.01926	1.0000
O	O279	1.0	0.77621	0.52971	0.01927	1.0000
O	O280	1.0	0.52621	0.52980	0.01924	1.0000
O	O281	1.0	0.89076	0.60624	0.01925	1.0000
O	O282	1.0	0.64081	0.60626	0.01928	1.0000
O	O283	1.0	0.76574	0.70339	0.01928	1.0000
O	O284	1.0	0.51585	0.70345	0.01935	1.0000
O	O285	1.0	0.65116	0.77980	0.01928	1.0000
O	O286	1.0	0.90121	0.77976	0.01931	1.0000
O	O287	1.0	0.76576	0.85622	0.01932	1.0000
O	O288	1.0	0.51582	0.85633	0.01926	1.0000
O	O289	1.0	0.18935	0.03673	0.93598	1.0000
O	O290	1.0	0.43942	0.03675	0.93595	1.0000
O	O291	1.0	0.30397	0.11319	0.93597	1.0000
O	O292	1.0	0.05391	0.11314	0.93597	1.0000

O	O293	1.0	0.18929	0.18950	0.93597	1.0000
O	O294	1.0	0.43939	0.18963	0.93595	1.0000
O	O295	1.0	0.06439	0.28669	0.93597	1.0000
O	O296	1.0	0.31432	0.28675	0.93595	1.0000
O	O297	1.0	0.17904	0.36313	0.93594	1.0000
O	O298	1.0	0.42895	0.36321	0.93598	1.0000
O	O299	1.0	0.06441	0.43949	0.93595	1.0000
O	O300	1.0	0.31439	0.43961	0.93598	1.0000
O	O301	1.0	0.26575	0.02286	0.85266	1.0000
O	O302	1.0	0.01581	0.02284	0.85263	1.0000
O	O303	1.0	0.14079	0.12013	0.85261	1.0000
O	O304	1.0	0.39077	0.12015	0.85262	1.0000
O	O305	1.0	0.27625	0.19653	0.85264	1.0000
O	O306	1.0	0.02610	0.19645	0.85258	1.0000
O	O307	1.0	0.14080	0.27283	0.85262	1.0000
O	O308	1.0	0.39079	0.27293	0.85264	1.0000
O	O309	1.0	0.01583	0.37007	0.85259	1.0000
O	O310	1.0	0.26576	0.37014	0.85264	1.0000
O	O311	1.0	0.15113	0.44651	0.85265	1.0000
O	O312	1.0	0.40121	0.44655	0.85264	1.0000
O	O313	1.0	0.31439	0.45355	0.76931	1.0000
O	O314	1.0	0.06445	0.45347	0.76926	1.0000
O	O315	1.0	0.42895	0.02990	0.76932	1.0000
O	O316	1.0	0.17903	0.02988	0.76929	1.0000
O	O317	1.0	0.31429	0.10633	0.76930	1.0000
O	O318	1.0	0.06438	0.10627	0.76928	1.0000
O	O319	1.0	0.18932	0.20350	0.76930	1.0000

O	O320	1.0	0.43933	0.20349	0.76927	1.0000
O	O321	1.0	0.30397	0.27997	0.76932	1.0000
O	O322	1.0	0.05393	0.27984	0.76926	1.0000
O	O323	1.0	0.18937	0.35635	0.76927	1.0000
O	O324	1.0	0.43941	0.35634	0.76928	1.0000
O	O325	1.0	0.26574	0.03680	0.68597	1.0000
O	O326	1.0	0.01577	0.03676	0.68594	1.0000
O	O327	1.0	0.15117	0.11327	0.68596	1.0000
O	O328	1.0	0.40118	0.11329	0.68600	1.0000
O	O329	1.0	0.26574	0.18964	0.68597	1.0000
O	O330	1.0	0.01588	0.18961	0.68599	1.0000
O	O331	1.0	0.14083	0.28680	0.68595	1.0000
O	O332	1.0	0.39075	0.28680	0.68596	1.0000
O	O333	1.0	0.02622	0.36323	0.68593	1.0000
O	O334	1.0	0.27622	0.36331	0.68596	1.0000
O	O335	1.0	0.14084	0.43965	0.68592	1.0000
O	O336	1.0	0.39075	0.43967	0.68600	1.0000
O	O337	1.0	0.30406	0.44663	0.60264	1.0000
O	O338	1.0	0.05403	0.44659	0.60259	1.0000
O	O339	1.0	0.18935	0.02300	0.60263	1.0000
O	O340	1.0	0.43942	0.02299	0.60262	1.0000
O	O341	1.0	0.31430	0.12020	0.60261	1.0000
O	O342	1.0	0.06440	0.12017	0.60264	1.0000
O	O343	1.0	0.17898	0.19656	0.60260	1.0000
O	O344	1.0	0.42894	0.19656	0.60266	1.0000
O	O345	1.0	0.06440	0.27303	0.60262	1.0000
O	O346	1.0	0.31431	0.27300	0.60264	1.0000

0	0347	1.0	0.18940	0.37021	0.60260	1.0000
0	0348	1.0	0.43945	0.37019	0.60265	1.0000
0	0349	1.0	0.39071	0.45353	0.51934	1.0000
0	0350	1.0	0.14082	0.45353	0.51925	1.0000
0	0351	1.0	0.27617	0.02986	0.51930	1.0000
0	0352	1.0	0.02617	0.02989	0.51926	1.0000
0	0353	1.0	0.39075	0.10637	0.51929	1.0000
0	0354	1.0	0.14077	0.10634	0.51927	1.0000
0	0355	1.0	0.26577	0.20351	0.51928	1.0000
0	0356	1.0	0.01583	0.20356	0.51934	1.0000
0	0357	1.0	0.15116	0.27989	0.51926	1.0000
0	0358	1.0	0.40121	0.27990	0.51934	1.0000
0	0359	1.0	0.26578	0.35634	0.51929	1.0000
0	0360	1.0	0.01579	0.35642	0.51924	1.0000
0	0361	1.0	0.68935	0.03670	0.93593	1.0000
0	0362	1.0	0.93943	0.03668	0.93599	1.0000
0	0363	1.0	0.80399	0.11306	0.93595	1.0000
0	0364	1.0	0.55391	0.11320	0.93594	1.0000
0	0365	1.0	0.68929	0.18949	0.93595	1.0000
0	0366	1.0	0.93936	0.18953	0.93598	1.0000
0	0367	1.0	0.56439	0.28676	0.93595	1.0000
0	0368	1.0	0.81432	0.28664	0.93595	1.0000
0	0369	1.0	0.67906	0.36313	0.93590	1.0000
0	0370	1.0	0.92896	0.36307	0.93596	1.0000
0	0371	1.0	0.56448	0.43957	0.93591	1.0000
0	0372	1.0	0.81442	0.43947	0.93593	1.0000
0	0373	1.0	0.76577	0.02283	0.85263	1.0000

0	0374	1.0	0.51580	0.02288	0.85261	1.0000
0	0375	1.0	0.64078	0.12011	0.85260	1.0000
0	0376	1.0	0.89079	0.12007	0.85262	1.0000
0	0377	1.0	0.77626	0.19648	0.85262	1.0000
0	0378	1.0	0.52612	0.19649	0.85257	1.0000
0	0379	1.0	0.64081	0.27283	0.85260	1.0000
0	0380	1.0	0.89080	0.27283	0.85261	1.0000
0	0381	1.0	0.51582	0.37014	0.85260	1.0000
0	0382	1.0	0.76581	0.37008	0.85258	1.0000
0	0383	1.0	0.65121	0.44650	0.85260	1.0000
0	0384	1.0	0.90123	0.44644	0.85259	1.0000
0	0385	1.0	0.81442	0.45349	0.76926	1.0000
0	0386	1.0	0.56444	0.45350	0.76928	1.0000
0	0387	1.0	0.92895	0.02987	0.76931	1.0000
0	0388	1.0	0.67904	0.02985	0.76927	1.0000
0	0389	1.0	0.81430	0.10630	0.76928	1.0000
0	0390	1.0	0.56439	0.10627	0.76929	1.0000
0	0391	1.0	0.68933	0.20350	0.76929	1.0000
0	0392	1.0	0.93932	0.20344	0.76926	1.0000
0	0393	1.0	0.80400	0.27990	0.76927	1.0000
0	0394	1.0	0.55391	0.27990	0.76928	1.0000
0	0395	1.0	0.68937	0.35634	0.76925	1.0000
0	0396	1.0	0.93946	0.35627	0.76924	1.0000
0	0397	1.0	0.76579	0.03682	0.68595	1.0000
0	0398	1.0	0.51576	0.03676	0.68597	1.0000
0	0399	1.0	0.65116	0.11326	0.68597	1.0000
0	0400	1.0	0.90120	0.11328	0.68596	1.0000

0	0401	1.0	0.76575	0.18965	0.68593	1.0000
0	0402	1.0	0.51585	0.18962	0.68603	1.0000
0	0403	1.0	0.64082	0.28681	0.68596	1.0000
0	0404	1.0	0.89079	0.28677	0.68592	1.0000
0	0405	1.0	0.52620	0.36325	0.68595	1.0000
0	0406	1.0	0.77623	0.36331	0.68594	1.0000
0	0407	1.0	0.64083	0.43965	0.68591	1.0000
0	0408	1.0	0.89078	0.43964	0.68598	1.0000
0	0409	1.0	0.80406	0.44663	0.60264	1.0000
0	0410	1.0	0.55402	0.44658	0.60259	1.0000
0	0411	1.0	0.68935	0.02301	0.60263	1.0000
0	0412	1.0	0.93942	0.02303	0.60257	1.0000
0	0413	1.0	0.81429	0.12025	0.60257	1.0000
0	0414	1.0	0.56439	0.12017	0.60266	1.0000
0	0415	1.0	0.67896	0.19658	0.60261	1.0000
0	0416	1.0	0.92895	0.19659	0.60262	1.0000
0	0417	1.0	0.56437	0.27301	0.60266	1.0000
0	0418	1.0	0.81430	0.27302	0.60260	1.0000
0	0419	1.0	0.68939	0.37020	0.60260	1.0000
0	0420	1.0	0.93946	0.37018	0.60263	1.0000
0	0421	1.0	0.89069	0.45356	0.51933	1.0000
0	0422	1.0	0.64079	0.45353	0.51927	1.0000
0	0423	1.0	0.77615	0.02989	0.51928	1.0000
0	0424	1.0	0.52618	0.02986	0.51928	1.0000
0	0425	1.0	0.89076	0.10642	0.51926	1.0000
0	0426	1.0	0.64075	0.10634	0.51928	1.0000
0	0427	1.0	0.76575	0.20355	0.51924	1.0000

0	0428	1.0	0.51581	0.20350	0.51937	1.0000
0	0429	1.0	0.65114	0.27986	0.51926	1.0000
0	0430	1.0	0.90121	0.27995	0.51930	1.0000
0	0431	1.0	0.76575	0.35637	0.51926	1.0000
0	0432	1.0	0.51579	0.35636	0.51928	1.0000
0	0433	1.0	0.18937	0.53672	0.93598	1.0000
0	0434	1.0	0.43944	0.53677	0.93598	1.0000
0	0435	1.0	0.30397	0.61321	0.93600	1.0000
0	0436	1.0	0.05390	0.61312	0.93591	1.0000
0	0437	1.0	0.18929	0.68950	0.93598	1.0000
0	0438	1.0	0.43940	0.68962	0.93598	1.0000
0	0439	1.0	0.06439	0.78670	0.93596	1.0000
0	0440	1.0	0.31431	0.78672	0.93597	1.0000
0	0441	1.0	0.17902	0.86313	0.93596	1.0000
0	0442	1.0	0.42894	0.86320	0.93598	1.0000
0	0443	1.0	0.06441	0.93950	0.93598	1.0000
0	0444	1.0	0.31437	0.93957	0.93597	1.0000
0	0445	1.0	0.26576	0.52286	0.85266	1.0000
0	0446	1.0	0.01582	0.52281	0.85259	1.0000
0	0447	1.0	0.14080	0.62012	0.85261	1.0000
0	0448	1.0	0.39078	0.62014	0.85263	1.0000
0	0449	1.0	0.27624	0.69653	0.85264	1.0000
0	0450	1.0	0.02612	0.69646	0.85257	1.0000
0	0451	1.0	0.14078	0.77283	0.85261	1.0000
0	0452	1.0	0.39078	0.77291	0.85265	1.0000
0	0453	1.0	0.01579	0.87011	0.85262	1.0000
0	0454	1.0	0.26573	0.87012	0.85265	1.0000

0	0455	1.0	0.15111	0.94651	0.85267	1.0000
0	0456	1.0	0.40119	0.94650	0.85263	1.0000
0	0457	1.0	0.31439	0.95352	0.76932	1.0000
0	0458	1.0	0.06442	0.95348	0.76929	1.0000
0	0459	1.0	0.42895	0.52992	0.76932	1.0000
0	0460	1.0	0.17904	0.52987	0.76927	1.0000
0	0461	1.0	0.31430	0.60636	0.76930	1.0000
0	0462	1.0	0.06440	0.60626	0.76929	1.0000
0	0463	1.0	0.18931	0.70351	0.76930	1.0000
0	0464	1.0	0.43932	0.70347	0.76927	1.0000
0	0465	1.0	0.30395	0.77996	0.76934	1.0000
0	0466	1.0	0.05391	0.77989	0.76928	1.0000
0	0467	1.0	0.18936	0.85637	0.76927	1.0000
0	0468	1.0	0.43940	0.85631	0.76928	1.0000
0	0469	1.0	0.26578	0.53684	0.68595	1.0000
0	0470	1.0	0.01578	0.53676	0.68595	1.0000
0	0471	1.0	0.15116	0.61327	0.68596	1.0000
0	0472	1.0	0.40119	0.61329	0.68597	1.0000
0	0473	1.0	0.26572	0.68966	0.68596	1.0000
0	0474	1.0	0.01587	0.68961	0.68602	1.0000
0	0475	1.0	0.14079	0.78681	0.68596	1.0000
0	0476	1.0	0.39074	0.78678	0.68596	1.0000
0	0477	1.0	0.02619	0.86324	0.68595	1.0000
0	0478	1.0	0.27620	0.86328	0.68597	1.0000
0	0479	1.0	0.14080	0.93964	0.68595	1.0000
0	0480	1.0	0.39073	0.93966	0.68600	1.0000
0	0481	1.0	0.30405	0.94660	0.60266	1.0000

0	0482	1.0	0.05401	0.94658	0.60260	1.0000
0	0483	1.0	0.18938	0.52302	0.60260	1.0000
0	0484	1.0	0.43944	0.52300	0.60260	1.0000
0	0485	1.0	0.31429	0.62023	0.60258	1.0000
0	0486	1.0	0.06439	0.62016	0.60264	1.0000
0	0487	1.0	0.17895	0.69658	0.60262	1.0000
0	0488	1.0	0.42893	0.69656	0.60265	1.0000
0	0489	1.0	0.06438	0.77302	0.60264	1.0000
0	0490	1.0	0.31429	0.77300	0.60264	1.0000
0	0491	1.0	0.18937	0.87018	0.60264	1.0000
0	0492	1.0	0.43944	0.87018	0.60264	1.0000
0	0493	1.0	0.39071	0.95352	0.51935	1.0000
0	0494	1.0	0.14079	0.95353	0.51928	1.0000
0	0495	1.0	0.27618	0.52987	0.51928	1.0000
0	0496	1.0	0.02618	0.52987	0.51925	1.0000
0	0497	1.0	0.39076	0.60640	0.51926	1.0000
0	0498	1.0	0.14078	0.60633	0.51927	1.0000
0	0499	1.0	0.26574	0.70352	0.51927	1.0000
0	0500	1.0	0.01584	0.70354	0.51934	1.0000
0	0501	1.0	0.15114	0.77987	0.51926	1.0000
0	0502	1.0	0.40120	0.77990	0.51933	1.0000
0	0503	1.0	0.26574	0.85634	0.51931	1.0000
0	0504	1.0	0.01578	0.85641	0.51924	1.0000
0	0505	1.0	0.68942	0.53668	0.93594	1.0000
0	0506	1.0	0.93942	0.53667	0.93594	1.0000
0	0507	1.0	0.80402	0.61310	0.93595	1.0000
0	0508	1.0	0.55395	0.61321	0.93595	1.0000

O	O509	1.0	0.68932	0.68949	0.93597	1.0000
O	O510	1.0	0.93939	0.68953	0.93593	1.0000
O	O511	1.0	0.56441	0.78676	0.93596	1.0000
O	O512	1.0	0.81432	0.78665	0.93596	1.0000
O	O513	1.0	0.67907	0.86313	0.93593	1.0000
O	O514	1.0	0.92894	0.86310	0.93599	1.0000
O	O515	1.0	0.56444	0.93954	0.93592	1.0000
O	O516	1.0	0.81438	0.93947	0.93598	1.0000
O	O517	1.0	0.76582	0.52283	0.85260	1.0000
O	O518	1.0	0.51583	0.52289	0.85262	1.0000
O	O519	1.0	0.64081	0.62010	0.85260	1.0000
O	O520	1.0	0.89080	0.62008	0.85259	1.0000
O	O521	1.0	0.77626	0.69648	0.85262	1.0000
O	O522	1.0	0.52613	0.69651	0.85259	1.0000
O	O523	1.0	0.64081	0.77283	0.85261	1.0000
O	O524	1.0	0.89079	0.77285	0.85262	1.0000
O	O525	1.0	0.51581	0.87013	0.85260	1.0000
O	O526	1.0	0.76580	0.87008	0.85260	1.0000
O	O527	1.0	0.65117	0.94648	0.85262	1.0000
O	O528	1.0	0.90120	0.94650	0.85267	1.0000
O	O529	1.0	0.81441	0.95349	0.76931	1.0000
O	O530	1.0	0.56442	0.95348	0.76929	1.0000
O	O531	1.0	0.92897	0.52988	0.76930	1.0000
O	O532	1.0	0.67906	0.52984	0.76924	1.0000
O	O533	1.0	0.81431	0.60630	0.76926	1.0000
O	O534	1.0	0.56440	0.60627	0.76928	1.0000
O	O535	1.0	0.68932	0.70349	0.76929	1.0000

0	0536	1.0	0.93933	0.70345	0.76927	1.0000
0	0537	1.0	0.80400	0.77990	0.76928	1.0000
0	0538	1.0	0.55390	0.77988	0.76928	1.0000
0	0539	1.0	0.68937	0.85632	0.76926	1.0000
0	0540	1.0	0.93941	0.85628	0.76928	1.0000
0	0541	1.0	0.76579	0.53682	0.68593	1.0000
0	0542	1.0	0.51578	0.53677	0.68595	1.0000
0	0543	1.0	0.65117	0.61327	0.68596	1.0000
0	0544	1.0	0.90122	0.61329	0.68597	1.0000
0	0545	1.0	0.76575	0.68966	0.68593	1.0000
0	0546	1.0	0.51586	0.68963	0.68602	1.0000
0	0547	1.0	0.64082	0.78680	0.68596	1.0000
0	0548	1.0	0.89080	0.78679	0.68592	1.0000
0	0549	1.0	0.52620	0.86324	0.68595	1.0000
0	0550	1.0	0.77622	0.86329	0.68596	1.0000
0	0551	1.0	0.64083	0.93964	0.68596	1.0000
0	0552	1.0	0.89076	0.93965	0.68599	1.0000
0	0553	1.0	0.80405	0.94663	0.60264	1.0000
0	0554	1.0	0.55401	0.94658	0.60261	1.0000
0	0555	1.0	0.68938	0.52303	0.60260	1.0000
0	0556	1.0	0.93944	0.52302	0.60258	1.0000
0	0557	1.0	0.81430	0.62023	0.60258	1.0000
0	0558	1.0	0.56439	0.62016	0.60265	1.0000
0	0559	1.0	0.67896	0.69658	0.60260	1.0000
0	0560	1.0	0.92895	0.69658	0.60263	1.0000
0	0561	1.0	0.56437	0.77302	0.60266	1.0000
0	0562	1.0	0.81431	0.77301	0.60261	1.0000

O	O563	1.0	0.68941	0.87018	0.60264	1.0000
O	O564	1.0	0.93944	0.87020	0.60262	1.0000
O	O565	1.0	0.89068	0.95355	0.51932	1.0000
O	O566	1.0	0.64079	0.95352	0.51929	1.0000
O	O567	1.0	0.77616	0.52990	0.51928	1.0000
O	O568	1.0	0.52617	0.52986	0.51926	1.0000
O	O569	1.0	0.89076	0.60643	0.51924	1.0000
O	O570	1.0	0.64076	0.60634	0.51927	1.0000
O	O571	1.0	0.76575	0.70353	0.51926	1.0000
O	O572	1.0	0.51581	0.70351	0.51936	1.0000
O	O573	1.0	0.65115	0.77988	0.51928	1.0000
O	O574	1.0	0.90121	0.77993	0.51931	1.0000
O	O575	1.0	0.76575	0.85636	0.51928	1.0000
O	O576	1.0	0.51578	0.85635	0.51928	1.0000

Dielectric Constant

The static dielectric tensor used for the magnetite supercell calculations is:

$$\epsilon = \begin{bmatrix} 19.764 & 0 & 0 \\ 0 & 13.188 & 0 \\ 0 & 0 & 16.030 \end{bmatrix}$$

This tensor was used in the calculation of the electrostatic correction term for charged defect formation energies.

DFT Calculation Parameters

Table 1: Summary of DFT calculation parameters used for magnetite and hematite.

**Parameter**	**Description**
Exchange-Correlation Functional	PBE-GGA with U correction
Hubbard U	Dudarev approach with $U_{eff} = 4.1$ eV on Fe $3d$ orbitals
Pseudopotentials	Fe_pv, O
Spin Polarization	Included in all calculations
Energy Convergence criterion	10^{-4} eV
Force Convergence criterion	0.01 eV/Å
Plane-Wave Cutoff (Magnetite)	600 eV for final energy calculation, 450 eV for structural relaxations
Supercells (Magnetite)	224-atom monoclinic for isolated vacancies and 448-atom supercells for Fe-O divacancy
K-Point Sampling (Magnetite)	Gamma-only for structural relaxation; $3\times3\times2$ for static calculations for 224-atom and $2 \times 2 \times 2$ for 448-atom supercells
